# Antipsychotic exposure is an independent risk factor for breast cancer: A systematic review of epidemiological evidence

**DOI:** 10.3389/fonc.2022.993367

**Published:** 2022-12-15

**Authors:** Zheng Gao, Yin Xi, Hekai Shi, Jiyuan Ni, Wei Xu, Kaili Zhang

**Affiliations:** ^1^ Department of Cardiology, The First Affiliated Hospital of Hebei Medical University, Shijiazhuang, Hebei, China; ^2^ Department of Internal Medicine, The Second Affiliated Hospital of Hebei Medical University, Shijiazhuang, Hebei, China; ^3^ Department of Orthopedics, The Third Affiliated Hospital of Hebei Medical University, Shijiazhuang, Hebei, China; ^4^ Department Of Adenosurgery, The Second Affiliated Hospital of Hebei Medical University, Shijiazhuang, Hebei, China

**Keywords:** breast cancer, antipsychotics, risk factor, atypical antipsychotics, prolactin-increasing antipsychotics

## Abstract

**Background:**

The effect of antipsychotics on breast cancer remains controversial.

**Materials and methods:**

Embase, Scopus, PubMed, Web of Science, Cochrane Library, and Hebei Medical University Library were used for the literature search. Observational studies with original data for the effects of antipsychotics on breast cancer were used. Studies of bed quality, those with inadequate sample size, incomplete follow-up works, or studies that did not meet the criteria were excluded. Meta-analysis was performed using R version 4.1.2. The odds ratio (OR) and its 95% confidence interval (CI) were used to evaluate the proportion of breast cancer in different groups. To detect possible sources of heterogeneity, subgroup and meta-regression analyses were employed.

**Results:**

Pooled data from 11 relevant studies with 1,499,001 participants suggested that individuals exposed to antipsychotics were more likely to suffer from breast cancer than those who were not exposed (OR, 1.23; 95% CI, 1.04–1.47). No significant difference in breast cancer prevalence between the atypical and typical antipsychotic groups was found (OR, 1.23; 95% CI, 0.93–1.63). Prolactin (PRL)-increasing and PRL-sparing antipsychotics posed a similar risk of breast cancer (OR, 1.13; 95% CI, approximately 0.97–1.31). Furthermore, the use of antipsychotics is attributed to increased mortality in patients with breast cancer (OR, 1.54; 95% CI, 1.29–1.82). Those exposed to antipsychotics at the maximum dose were more likely to suffer from breast cancer than those exposed to the minimum dose.

**Conclusions:**

Antipsychotic exposure is an independent risk factor for breast cancer. No significant difference in the risk of breast cancer between typical and atypical antipsychotics was noted. Those exposed to antipsychotics at higher doses are more likely to suffer from breast cancer. Moreover, the use of antipsychotics is attributed to increased mortality in patients with breast cancer. PRL-increasing and PRL-sparing antipsychotics pose a similar risk of breast cancer.

**Systematic review registration:**

https://www.crd.york.ac.uk/PROSPERO/, identifier CRD42022307624.

## Introduction

Increased health awareness, effective prevention strategies, and improved access to medical treatment are extremely significant to curb the increasing global burden of breast cancer, particularly in the most affected countries (1). Furthermore, taking antipsychotic drugs is a common treatment for mental illness. Some researchers believe that some antipsychotics increase the risk of breast cancer by increasing serum PRL levels, as PRL stimulates the proliferation of breast tumors (2–5). Moreover, the antidopaminergic mode of action was believed to be the critical mechanism (6), suggesting that antipsychotics counteract the tonic inhibitory effect of dopamine on PRL secretion, which results in PRL secretion disinhibition (7). Pituitary PRL is primarily regulated by dopamine (8), whereas extrapituitary PRL is insensitive to this neurotransmitter (9). This explains why dopamine agonists, including bromocriptine, are ineffective in patients with PRL-dependent breast cancer (10). Therefore, the effect of antipsychotics is believed to be mainly through affecting the pituitary PRL level. The introduction of second-generation antipsychotics, also known as atypical antipsychotics, has been a revolutionary pharmacological step for treating psychotic illness. The second-generation antipsychotics are believed to be with less extrapyramidal effects (11). Differences between typical and atypical antipsychotics exist, including differences in target receptors and metabolic differences (12). Although all antipsychotics have the propensity to induce hyperprolactinemia (HPRL), typical antipsychotics are believed to be more associated with sustained HPRL. They have a higher affinity for dopamine receptor (DR) and slower dissociation from the receptor once bound. However, atypical antipsychotics are relatively different in causing high PRL levels. Most atypical antipsychotics do not cause an increase in the PRL level, except for a few drugs, including risperidone, 9-hydroxyrisperidone, and amisulpride. This may be associated with the higher peripheral-to-central dopamine receptor potency of either the parent drug or its active metabolite (13). However, the conclusions of different studies remain controversial. Several previous studies have identified possible pathways through which antipsychotics promote breast cancer (14, 15). In contrast, other studies have found that some antipsychotic drugs or antipsychotics that induced HPRL can prevent the growth or metastasis of breast tumor cells (16, 17). However, the conclusions of different clinical trials also remain controversial. Additionally, studies that have conclusively summarized the effects of different antipsychotics and the role of PRL are lacking. We performed this systematic review and meta-analysis to determine the relationship between the use of antipsychotics and the risk of breast cancer. Moreover, we aimed to explore the differences between the first and second generations of antipsychotics and the role of PRL.

## Material and methods

We followed the Preferred Reporting Items for Systematic Reviews and Meta-analyses (PRISMA) (18) and Meta-analysis of Observational Studies in Epidemiology (19) reporting guidelines. The present review has been registered in the International Prospective Register of Systematic Reviews (https://www.crd.york.ac.uk/PROSPERO/, CRD42022307624). All the included studies were confirmed to be free of ethical and moral concerns. Data for the review were anonymously analyzed. Therefore, the ethics committee waived the need for consent.

### Search strategy

The online databases included Embase, Scopus, PubMed, Web of Science, and Cochrane Library. The Hebei Medical University Library was browsed for related books and documents with the administrators’ help. The literature search was performed on 19 December 2021, without language and date restrictions. The search terms and strategy were described in the **Supplementary Material S1**, Appendix. Relevant articles were further screened manually for those interested literature.

### Eligibility criteria

We excluded studies that may lead to an imprecise conclusion. Retrospective or prospective cohort studies and case–control studies were accepted. Due to the lack of relevant results, we excluded randomized controlled trials (RCTs), as introduced later. The difference in study design may bring uncertainty to the evidence provided in this study. The study type should be clearly defined. Observational studies should introduce the sources of epidemiological data. Sources, including hospital medical records, public health databases, and insurance agencies, were acceptable. Case−control studies should provide a clear selection criterion for the populations in control groups. Moreover, the baseline characteristics of the control groups should be strictly the same as those of the case groups. Participants in each group should be sufficient to avoid contingency, which means at least 100 participants for each group. Follow-up work should be completed, ensuring that all data are available. Criteria for breast cancer or antipsychotics should be clearly defined, which should be consistent with universal knowledge. Studies that received “critical risk” overall during quality assessments were excluded to avoid evidence of bad credibility. Patients with primary HPRL not caused by psychotropics at the beginning of the follow-up work should be excluded. Moreover, in cases of metastatic cancer unrelated to psychotropics, patients with multiple immune diseases caused by perimenopausal endocrine disorders should be excluded. Non-population-based virtual statistical models were not accepted. Review articles, case reports, or protocols were not applied in the present systematic review.

### Data extraction

Three reviewers independently performed the literature identification (GZ, XY, and SH), under the direction of an epidemiologist (XW). Any disagreement was reported to XW. All the data were directly extracted from the literature. We recorded the number of breast cancers patients. The number of antipsychotics use and the types and dosage of antipsychotics were also recorded. We further extracted the characteristics of studies, including age at enrollment, follow-up duration, psychiatric diagnosis, the basis for grouping, and the country and region in which the trial was conducted. Details are described in **Table 1**.

**Table 1:** Baseline characteristics of the included studies.

Study	Total	Mean age at entry a (years)	Mean Follow-up (years)	Psychiatric diagnosis			Location		Group biases
				Schizophrenia	Other	Never			
**SO Dalton—2006** (20)	474,247	58.7	6.4	1,547	7788	15,929	Denmark	Retrospective cohort study	b
**George—2020**(21)	155,737	63.19	14.8	NR	NR	NR	America	Retrospective cohort study	b.c
**Ana Isabel Wu Chou—2017 **(22)	21,454	41.7	NR^a^	10,727	NR	NR	Taiwan	Retrospective cohort study	b
**Kato—2000 **(23)	15,270	NR	7.3	NR	NR	NR	New York	Prospective cohort study	b
**Anton—2018 **(2)	662,320	62	NR	2,998	50196	NR	Denmark	Case−control study	b
**Laurent Azoulay—2011 **(24)	11,240	66.8	7.8	NR	NR	NR	UK	Retrospective cohort study	c
**Tae Maeshima—2021**(25)	16,230	NR	NR	NR	NR	NR	Japan	Retrospective cohort study	c
**Johan Reutfors—2016 **(26)	55,976	59.6	2.6	NR	NR	NR	Swedish	Retrospective cohort study	c
**Heidi Taipale—2021**(27)	2,493	62.2	6.5	NR	NR	NR	Finland	Case−control study	b.d
**Judith P. Kelly—1999**(28)	11,628	53	NR	NR	NR	NR	America	Case−control study	b
**Julia Hippisley−Cox—2015 **(29)	60,609	61	NR	49	NR	10,444	America	Case−control study	b
**Blánaid M. Hicks—2020 **(30)	NR	63.6	NR	NR	NR	NR	UK	Cohort study	e

a NR means not reported.

b Whether to receive antipsychotic medication.

c Typical antipsychotic drugs compared with atypical antipsychotic drugs.

d Different doses of prolactin-increasing drugs in schizophrenia patients.

e Breast-cancer-specific mortality comparing the use of antipsychotics with non-use.

### Evaluation of study quality and risk of bias

Quality assessment for each study was performed using the risk of bias in non-randomized studies of interventions (31) in R version 4.1.2 software. One reviewer (GZ) evaluated all the studies to be included in the following seven domains: bias due to confounding, bias due to the selection of participants, bias in the classification of interventions, bias due to deviations from intended interventions, bias due to missing data, bias in measurement of outcomes, and bias in the selection of the reported results. Each risk was labeled “low risk,” “moderate risk,” “serious risk,” and “critical risk.” The overall risk was manually evaluated following the criteria set previously: studies with two domains of “moderate risk” or one domain of higher risk were believed to be at “moderate risk” and studies with three or more risks and one or more “serious risks” were believed to be at “serious risk.” Studies with two or more “critical risks” were excluded. Details of quality evaluation are presented in **Supplementary Figures S1A, B**.

### Statistical analysis

Data synthesis and statistical analyses were performed by one reviewer (GZ) and double checked by another (XY).

Odds ratios (ORs) and their 95% confidence intervals (CIs) were used to evaluate the proportion of breast cancer in different groups for the meta-analyses. To detect possible sources of heterogeneity, subgroup analysis and meta-regression analysis were employed. All statistical effects were calculated using a random-effect model. A two-tailed α = 0.05 was set as the statistical significance. The I^2^ test was calculated as a measure of heterogeneity, in which I^2^ values of 25%, 50%, and 75% indicate low, moderate, or high heterogeneity, respectively (32). We considered heterogeneity during the evaluation of the effect.

All statistical analyses were performed in R version 4.1.2 using the package “meta” (R Project for Statistical Computing) (R Core Team. R: a language and environment for statistical computing. Vienna R Foundation for Statistical Computing; 2019. https://www.R-project.org).

Peter’s test and Deek’s funnel plots were used to detect potential publication bias. The included studies were excluded one by one for sensitivity analysis for each outcome. Potential publication bias was corrected using “trim and fill” (33). Potential publication bias was suggested to be significant if corrected results challenged the previous conclusions.

### Outcomes and comparisons

We compared the proportion of breast cancer between those who never and ever used antipsychotics. Furthermore, the effects of typical and atypical antipsychotics were compared. We compared the prevalence of breast cancer in the PRL-increasing or PRL-sparing antipsychotic groups to assess the role of PRL in the effect of antipsychotics on breast cancer development. We further evaluated the risk of breast cancer in individuals exposed to antipsychotics of a higher or lower dose. Additionally, breast cancer mortality was assessed in the antipsychotic-exposed or antipsychotic-unexposed groups.

## Results

### Literature identification

**Figure 1:**
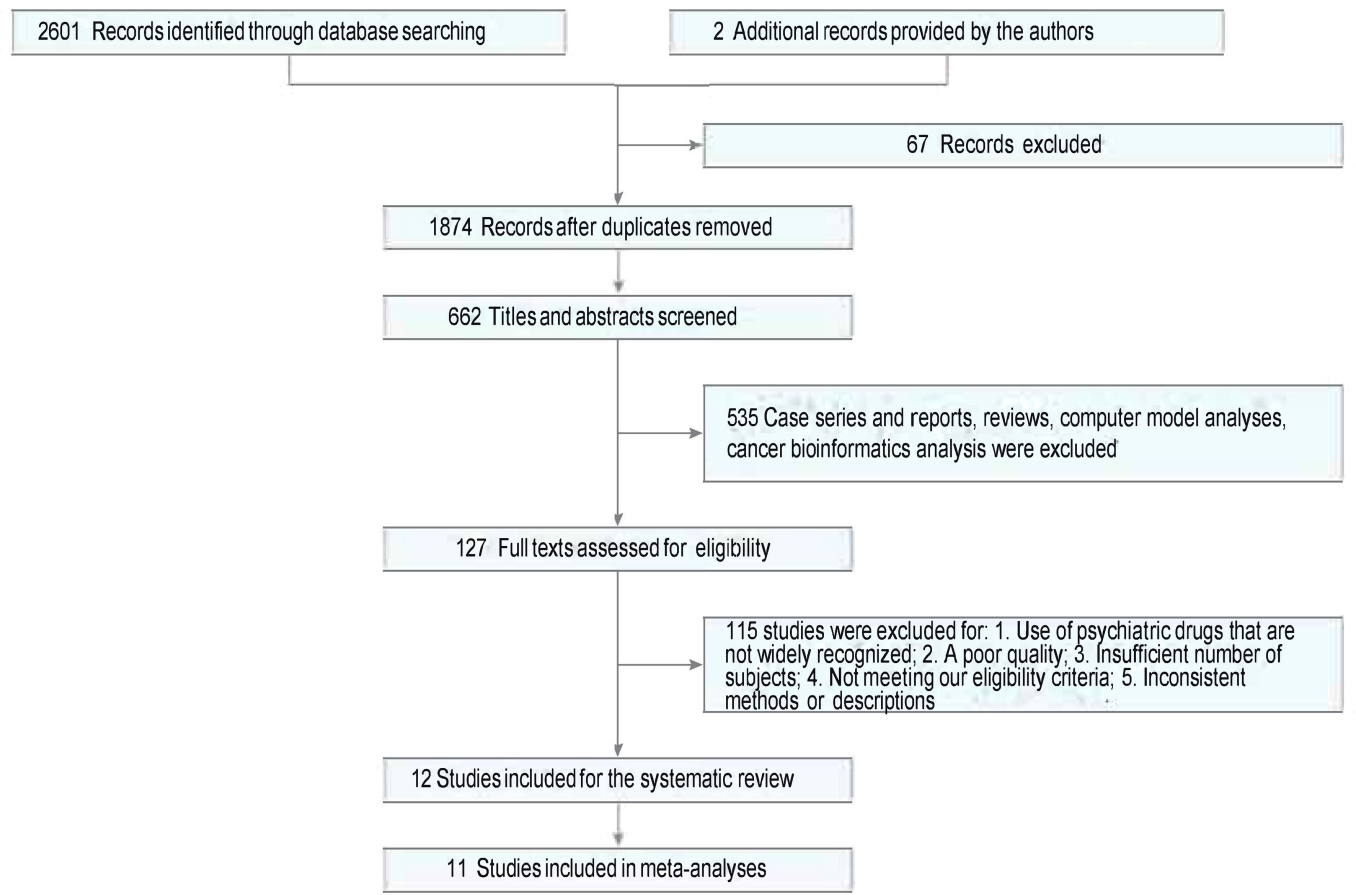
PRISMA flow diagram summarizing the article selection process.

Twelve studies were identified from 2,603 studies for a systematic review of the present evidence. Eleven studies with 1,499,001 participants were included in the meta-analysis. The flow chart summarizing the literature search and identification is shown in **Figure 1**. Of the 11 observational studies (2, 20–29), six (54.55%) were retrospective studies (20–22, 24–26) (involving 734,884 individuals), one (9.09%) was a prospective cohort study (23) (involving 15,270 individuals), and four (36.36%) were case−control studies (2, 27–29) (involving 748,847 individuals). One (9.09%), two (18.18%), and eight studies (66.67%) had “serious risk,” “moderate risk,” and “low risk,” respectively, during the quality evaluation. Studies that were “critical risk” overall were excluded. In seven studies, the prevalence of breast cancer was compared between those exposed to antipsychotics and those who were not. Two studies (2, 27) evaluated the prevalence of breast cancer exposed to antipsychotics in a high or low cumulative dose or defined daily doses (DDDs). The effects of different antipsychotics (typical or atypical) were compared in four studies (24–27). The mean age at entry (years) was similar in both groups except for Ana Isabel Wu Chou–2017 (22). The mean follow-up (years) was similar in both groups except for a study by Johan Reutfors in 2016 (26). Baseline characteristics are described in **Table 1**.

### Data synthesis

#### The effect of antipsychotics

**Figure 2:**
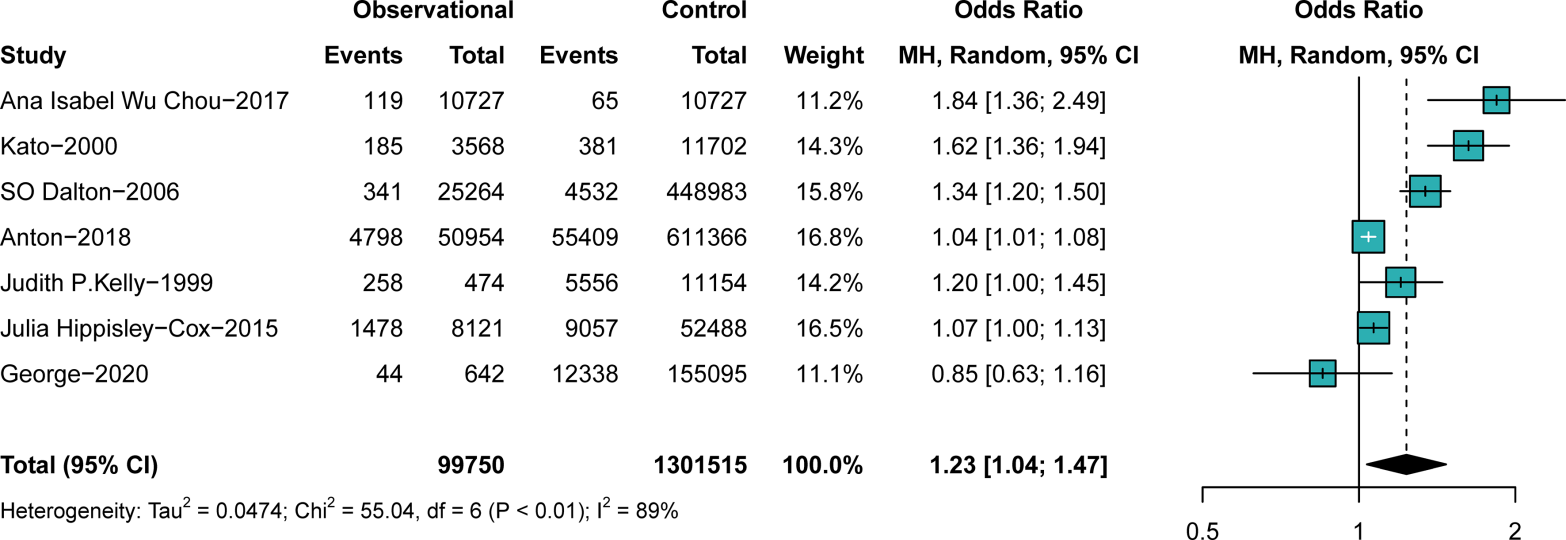
The difference in the prevalence of breast cancer between those exposed to antipsychotics or not. Point sizes are an inverse function of the precision of the estimates, and bars correspond to 95% CIs.

In comparison A (participants exposed to antipsychotics versus those not exposed to antipsychotics, n = 1,401,265), participants exposed to antipsychotics were at a higher risk of breast cancer than those not exposed to antipsychotics (OR, 1.23; 95% CI, 1.04–1.47), with significant heterogeneity (I^2^ = 89%, p < 0.01), as shown in the forest plot in **Figure 2**. No obvious publication bias was detected. Study designs resulted in no significant heterogeneity, as shown in the subgroup analysis (**Supplementary Figure S2**). The nationality of participants makes some difference in both the outcome and the heterogeneity, as shown in **Supplementary Figure S3**. Studies performed in Denmark (OR, 1.18; 95% CI, 0.92–1.51) and the United States (US) (OR, 1.17; 95% CI, 0.91–1.50) suggested insignificant effects of antipsychotics on breast cancer. The prevalence difference of breast cancer between groups became insignificant when some studies were excluded (**Supplementary Figures S4, S5**). Individuals’ age at entry (years) (QM = 10.8493, p = 0.001) was considered a vital source of heterogeneity, whereas the mean follow-up (years) (QM = 1.0480, p = 0.3060) resulted in insignificant heterogeneity (**Supplementary Figures S6–S9**). We further performed a subgroup analysis to explore the difference between random drug use and drugs used in a mentally ill population. The result suggests that precise drug use in psychiatric patients is more likely to cause breast cancer than random drug use (OR _schizophrenia_, 1.84; 95% CI, 1.36–2.49; subgroup differences, p < 0.01) (**Supplementary Figure S10**), as indicated by the sensitivity analysis of comparison A.

**Figure 3:**
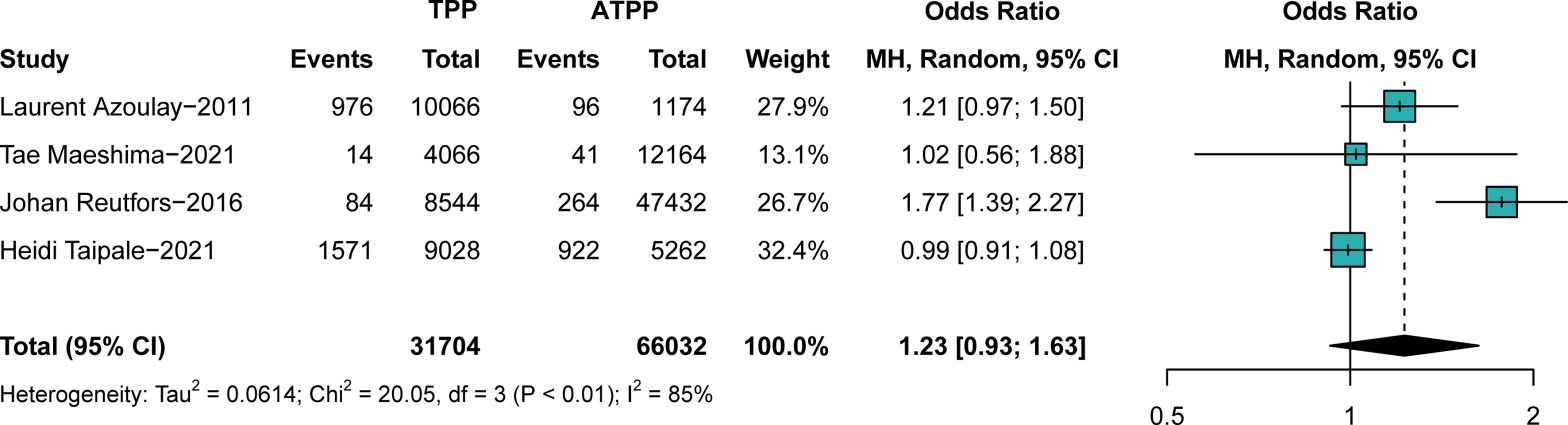
The difference in the prevalence of breast cancer between those exposed to typical antipsychotics or atypical antipsychotics. Point sizes are an inverse function of the precision of the estimates, and bars correspond to 95% CIs. TPP, typical antipsychotics; ATPP, atypical antipsychotics.

In comparison B (participants exposed to typical antipsychotics versus those exposed to atypical antipsychotics, n = 97,736), no significant difference in breast cancer prevalence was found in different groups (OR, 1.23; 95% CI, 0.93–1.63), with significant heterogeneity (I^2^ = 85%, p < 0.01), as shown in the forest plot in **Figure 3**. No obvious publication bias was detected. As shown in the subgroup analyses (**Supplementary Figures S11, S12**), the study design and continent were not sources of heterogeneity. The outcome was stable when studies were excluded one by one (**Supplementary Figures S13, S14**). Associations between antipsychotics and breast cancer were more substantial in younger individuals, particularly in those younger than 45 years old. Publication biases of comparisons A and B are described in **Supplementary Figures S15–S18**. The subgroup analysis indicates that the association was stronger in female Chinese populations.

#### Secondary outcomes

**Figure 4:**
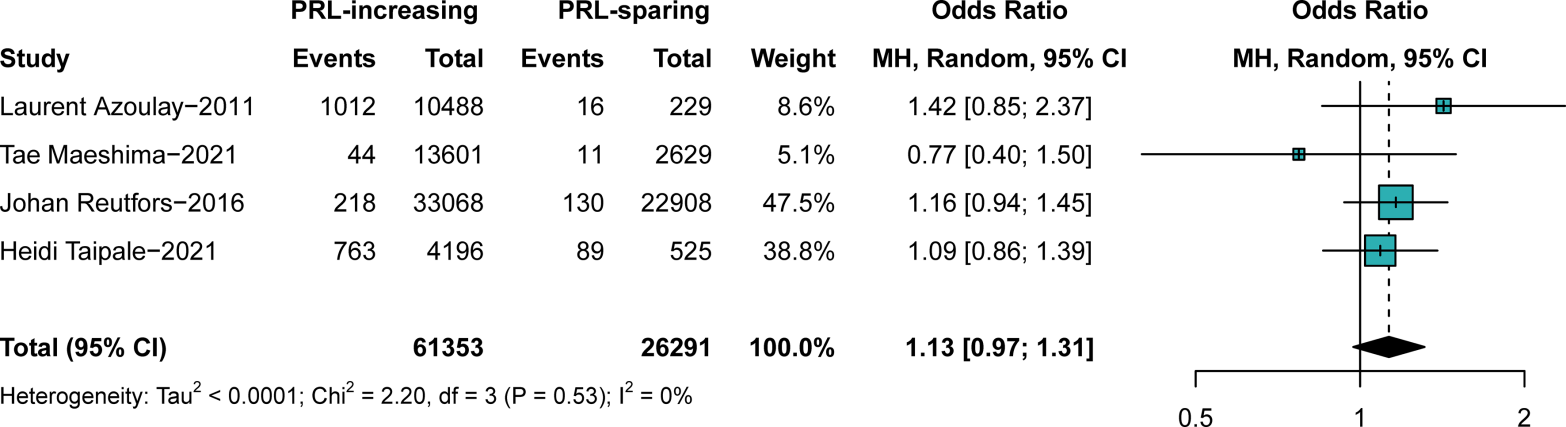
The difference in the prevalence of breast cancer between those exposed to prolactin-increasing or prolactin-sparing antipsychotics. Point sizes are an inverse function of the precision of the estimates, and bars correspond to 95% CIs. PRL, prolactin.

We further investigated the effects of PRL-increasing and PRL-sparing antipsychotics on breast cancer prevalence. We aimed to confirm whether PRL plays a crucial role in the pathophysiology of antipsychotic-induced breast cancer. In comparison C, patients with all the typical antipsychotics and PRL-increasing atypical antipsychotics for more than 5 years were assigned to the observational groups. Patients with other atypical antipsychotics for more than 5 years were assigned to the control groups. The results suggested that PRL-increasing and PRL-sparing antipsychotics pose a similar risk of breast cancer (OR, 1.13; 95% CI, approximately 0.97–1.31), with very low heterogeneity, as shown in **Figure 4**. No obvious publication bias was detected (**Supplementary Figures S19, S20**). The outcome was stable when studies were excluded one by one (**Supplementary Figures S21, S22**).

The studies by Taipale (27) (OR, 1.39; 95% CI, 1.16–1.67) and Anton (2) (OR, 1.33; 95% CI, 1.15–1.55) indicated that those exposed to antipsychotics at the maximum dose are more likely to suffer from breast cancer than those exposed to the minimum dose. Moreover, we examined all-cause mortality in the available studies. Data from the study by Hicks (30) suggested higher all-cause mortality in patients with cancer who were exposed to antipsychotics (OR, 1.54; 95% CI, 1.29–1.82). A summary of our findings is provided in **Table 2**. The results of the quality assessments and sensitivity analysis suggest moderately good credibility of the evidence for this review.

**Table 2:** Summary of findings.

Outcomes	Number of studies	Number of participants	Pooled results			
			OR	(95%CI)		heterogeneity
						I^2^(%)
**Antipsychotics *vs*. non-antipsychotics groups**	7	1,401,265	1.23	1.04	1.47	89%
**Typical *vs*. atypical antipsychotics groups**	4	97,738	1.23	0.93	1.63	86.00%
**All cause motility**	1	23,695	1.54	1.29	1.82	
**Subgroup analyses of breast cancer risk in the antipsychotics and non-antipsychotics groups**						
**Cohort study**	4	666,708	1.37	1.01	1.86	82%
**Case−control study**	3	734,557	1.05	1.02	1.08	21%
**China**	1	21,454	1.84	1.36	2.49	N
**United States**	4	243,244	1.17	0.91	1.50	87%
**Denmark**	2	1,136,567	1.18	0.92	1.51	95%
**Subgroup analysis of the breast cancer risk in typical and atypical antipsychotics groups**						
**Cohort study**	3	83,446	1.16	0.95	1.40	3%
**Case−control study**	1	14,290	1.09	0.86	1.39	N
**Europe**	3	81,504	1.15	0.99	1.34	0%
**Asia**	1	16,230	0.77	0.40	1.50	N
**High-dose groups *vs*. low-dose groups**						
**Heidi Taipale’s study**	1	41375	1.39	1.16	1.67	N
**Anton’s study**	1	1739	1.33	1.15	1.55	N

OR, odds ratio; CI, confidence interval; N, not applicable.

## Discussion

The present systematic review and meta-analysis investigated epidemiological evidence about breast cancer and antipsychotics. Data from 11 observational studies of 1,499,001 individuals indicated that antipsychotics are an independent risk factor for breast cancer and suggested no significant difference in the effect of antipsychotics of different generations. Particularly, the associations were stronger in younger individuals (younger than 45 years old). The association was stronger for female Chinese populations. Moreover, our results revealed that individuals exposed to antipsychotics at the minimum dose are more likely to suffer from breast cancer than those exposed to the maximum dose. Furthermore, the use of antipsychotics is attributed to increased mortality in patients with breast cancer. Those taking PRL-increasing and PRL-sparing antipsychotics for more than 5 years showed no difference in the risk of breast cancer. It is worth noting that typical antipsychotics were generally reported to cause an increase in PRL. Only some of the atypical antipsychotics, including risperidone, 9-hydroxyrisperidone, and amizupired, have caused an increased PRL level.

To date, the present review is the first meta-analysis that comprehensively summarized the effects of antipsychotics on breast cancer and had the largest sample size. The studies by Taipale (27) and Anton (2) evaluated the prevalence of breast cancer among those exposed to antipsychotics in different cumulative doses or DDDs, which we considered in the systematic review although not for meta-analysis owing to the inconsistent group bias. In previous studies, some have reported the association between PRL and antipsychotics and suggested antipsychotics as a potential risk factor for breast cancer; however, they failed to provide a confirming conclusion (34). Our review helps to determine the association between antipsychotics and breast cancer and the role of PRL. Furthermore, the conclusions of the present meta-analysis were consistent with those of a recent observational study conducted in the US (35).

A large meta-analysis by Correll (36) showed that patients with schizophrenia had a significantly higher etiology-specific mortality than the general population. The study by Correll was the first large-scale meta-analysis of schizophrenia, opening the way to study all-cause mortality in schizophrenia and concluding that schizophrenia is associated with several diseases. However, the study did not rigorously analyze the factors that influence breast cancer and the occurrence of each disease. A review by Leung (37) also suggested that antipsychotics are associated with breast cancer and hypothesized that the longer antipsychotic drugs are used, the higher the incidence of breast cancer. In our study, we consolidated the two findings. We directed the relationship between the first and second generations of antipsychotics and breast cancer. Additionally, we analyzed the association of antipsychotics that increased the PRL level with breast cancer and obtained the following conclusions: no significant difference in breast cancer risk between typical and atypical antipsychotics was noted, and PRL-increasing and PRL-sparing antipsychotics pose a similar risk of breast cancer.

The study by Anna George (21) only enrolled postmenopausal female individuals, which showed a critically inconsistent effect of antipsychotics. This may be associated with different PRL levels in pre- and postmenopausal women. As a polypeptide hormone with a wide range of physiological functions, PRL is mainly secreted and synthesized by the pituitary gland. It has been reported that PRL plays a significant role in the proliferation of breast tissues and normal malignant tumor tissues, including enhancing the migration and proliferation of tumor cells, angiogenesis, sand apoptosis (38, 39). In the studies by Laurent (24), Tae (25), and Johan (26), risperidone was singled out for comparison. The researchers believed that risperidone differs from other atypical antipsychotics due to its PRL-sparing effect. Therefore, in our study, risperidone-taking patients were included in the observation group in comparison C, along with patients using other PRL-increasing atypical antipsychotics and those with any typical antipsychotics.

The pathogenesis and risk factors for breast cancer remain unclear (40). Molecules and targets have been detected and applied in clinical practice and drug development, most of which are associated with the endocrine system. Classical ones include the estrogen receptor proposed by Beatson in 1896 (41), human epithelial growth factor receptor-2 (42, 43), and progesterone receptor. Recent studies have suggested that PRL receptors are more highly expressed in breast cancer tissues and cells (44) and may play a significant role in the mechanism of endocrine resistance in breast cancer therapies (45). The results of some *in vitro* cytological studies and animal experiments corroborate this view (46). However, our review suggests no significant difference in breast cancer risk between PRL-increasing and PRL-sparing antipsychotics. One potential reason is that studies on the relationship between PRL levels and breast cancer risk have only focused on circulating PRL levels. However, PRL is not only secreted by the pituitary gland and used in distal tissues, including the mammary gland, but also acts in an autocrine/paracrine manner. Additionally, PRL is produced by breast (tumor) cells and acts directly on the cell itself (autocrine) or adjacent cells (paracrine) (47). We suggest that there may be other ways wherein antipsychotics increase the risk of breast cancer.

The dominant mechanism of antipsychotics’ effect on PRL levels was considered to inhibit dopamine D2 receptor on PRL cells in the anterior pituitary. Thus, the inhibition of dopamine on PRL cells was decreased. Antipsychotic-induced HPRL may lead to multisystem adverse reactions, including reproductive, sexual, and immune dysfunctions (48). It has been reported that antipsychotics of different types or dosages may diversely affect PRL levels (49, 50). Conversely, the present meta-analysis did not suggest a significant difference between typical and atypical antipsychotics. It is worth noting that breast cancer rates were particularly higher for paliperidone-, sulpiride-, and risperidone-exposed individuals, which is consistent with previously reported results (49).

The pooled result changed in comparison A when some studies were excluded, as shown in the sensitivity analysis. The main cause was considered inconsistent in the population. In the study by Isabel (22), the mean age at entry was 41.7 years old, which was at least 10 years younger than those of others. Therefore, the difference in female endocrine status may be one of the main sources of uncertainty and heterogeneity in the pooled result (51). There were fewer individuals or cases enrolled in the study by Kato (23) than those in other case−control or cohort studies, which is also considered a cause of heterogeneity. Neuroleptic medicines, including phenothiazines, thiozanthenes, butyrophenones, diazepin–oxazepines, benzamides, diphenylbutylpiperidines, and indoles, were contained in the cohort of Dalton (20). The variety of regimens could bring heterogeneity into the meta-analysis. Antidepressant use was not adjusted for in the study by Kelly (28) when comparing breast cancer risk in different groups, which may affect the result and cause heterogeneity. Furthermore, we have no detailed information on individuals’ antipsychotic use during the follow-up years. All of these factors may have influenced the outcomes.

The present review had several limitations. The large amount of unexplained heterogeneity across studies was the most notable. This is likely attributed to variability in populations (age at entry or races), variability in study designs (including the source of data and follow-up duration), measurement tools, sociodemographic factors, frequency and type of testing, local policies, and natural environment. The variability in antipsychotics was another notable source of heterogeneity. As mentioned above, the growth of breast tumor tissue is affected by both pituitary PRLs and paracrine/autocrine PRLs. However, paracrine/autocrine PRL varies greatly among different patients. If PRL plays a key role in antipsychotics and breast carcinogenesis, autocrine and paracrine PRL may introduce considerable heterogeneity into our study. Moreover, the type and dose of antipsychotic drugs varied widely among the included studies, which may have introduced heterogeneity.

In comparison A, the significant difference became insignificant when some studies were excluded. Although sources of this uncertainty or heterogeneity seem to be detected, the conclusion of this review should be explained with caution. Several studies have reported potential associations of mental disorders with the risk of breast cancer incidence and its recurrence or mortality (52, 53). We performed a subgroup analysis for comparison A and found that the effect of antipsychotics was more significant in studies that included psychiatric patients as an observational group. This suggests that mental illness may affect the results of the present review. In comparisons B and C, antipsychotics were used in all the included populations; however, it was difficult to know whether the participants suffered from psychosis. Therefore, we failed to accurately adjust the effects of mental disease on the drugs. This may be possibly due to a lack of specific individuals. This may raise the potential confounding bias of the present meta-analysis. Thus, the conclusion of the present review should be considered more cautiously. Regarding the variety of species, most studies have not focused on a concrete one or described the percentage use of different antipsychotics, indicating that the effect of different antipsychotics could not be clearly identified. The present review is a synthesis of data from observational studies. Due to various reasons, including ethnic prohibition, RCTs were insufficient for a more credible synthetic conclusion.

Questions remain regarding breast cancer and antipsychotics. The most significant among them is the lack of RCTs or cohort studies reporting changes in the prevalence of breast cancer following antipsychotic treatment termination. Data were unavailable to confirm the effect of antipsychotic treatment termination or PRL lowering on the prevalence of breast cancer, which requires more studies. Due to the autocrine/paracrine of PRL, although the pooled data suggest that PRL-increasing and PRL-sparing antipsychotics pose a similar risk of breast cancer, we cannot deny the role of PRL in breast cancer. Studies are needed to accurately evaluate the effects of autocrine/paracrine PRL on breast cancer and antipsychotic drug-induced breast cancer.

## Conclusions

Our review demonstrated that antipsychotic exposure is an independent risk factor for breast cancer. Moreover, no significant difference in the risk of breast cancer between typical and atypical antipsychotics was noted. In addition, those exposed to antipsychotics at higher doses are more likely to suffer from breast cancer. Moreover, the use of antipsychotics is attributed to increased mortality in patients with breast cancer. Lastly, PRL-increasing and PRL-sparing antipsychotics pose a similar risk of breast cancer.

## Data availability statement

The original contributions presented in the study are included in the article/**Supplementary Material**. Further inquiries can be directed to the corresponding author.

## Author contributions

KZ led the study and contributed to team management, revised the manuscript, polished the language, and contributed to drafting the articles or critical revision for important intellectual content. KZ approved the final version to be published and agreed to be accountable for all aspects of the work in ensuring that questions related to the accuracy or integrity of any part of the article are appropriately investigated and resolved. ZG contributed to the data analysis and manuscript formatting. ZG, YX and HS contributed to the literature search, study design and identification, data acquisition, and recording of the characteristics of the studies. WX evaluated the quality of the observational studies and reviewed and rectified the data. ZG, YX, HS, WX, and JN contributed to the data interpretation and critical revision of the manuscript. HS embellished the images and further interpreted the data. KZ contributed to the literature downloading and contacted the literature authors. All authors contributed to the article and approved the submitted version.
