# Supplementary material for: Antipsychotic exposure is an independent risk factor for breast cancer: A systematic review of epidemiological evidence
Source: Front Oncol. 2022 Dec 15;12:993367. doi: 10.3389/fonc.2022.993367 (PMC9798228; doi:10.3389/fonc.2022.993367)
Supplement: Supplementary file 1 [file DataSheet_1.docx]

**Supplements**

A


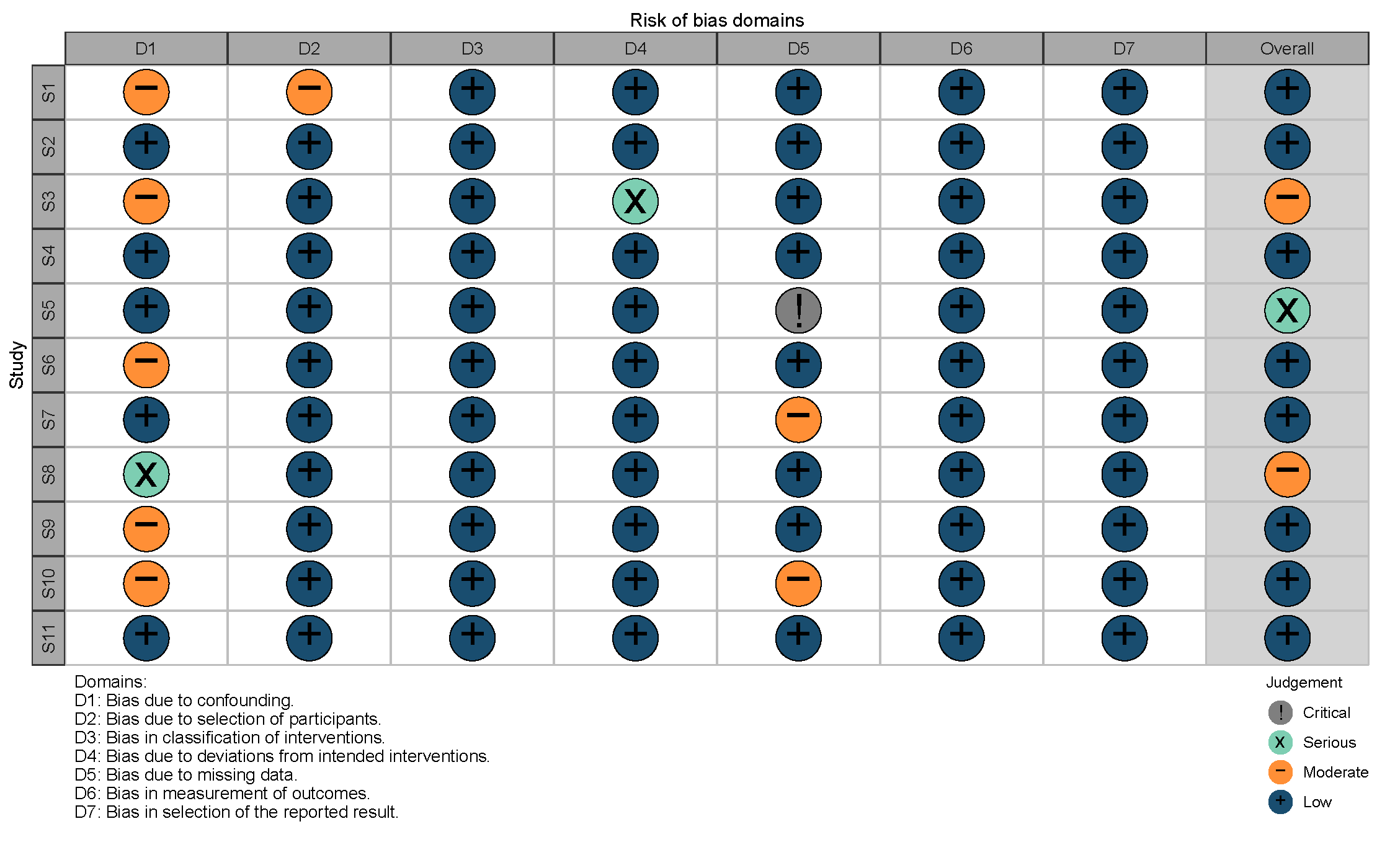


B
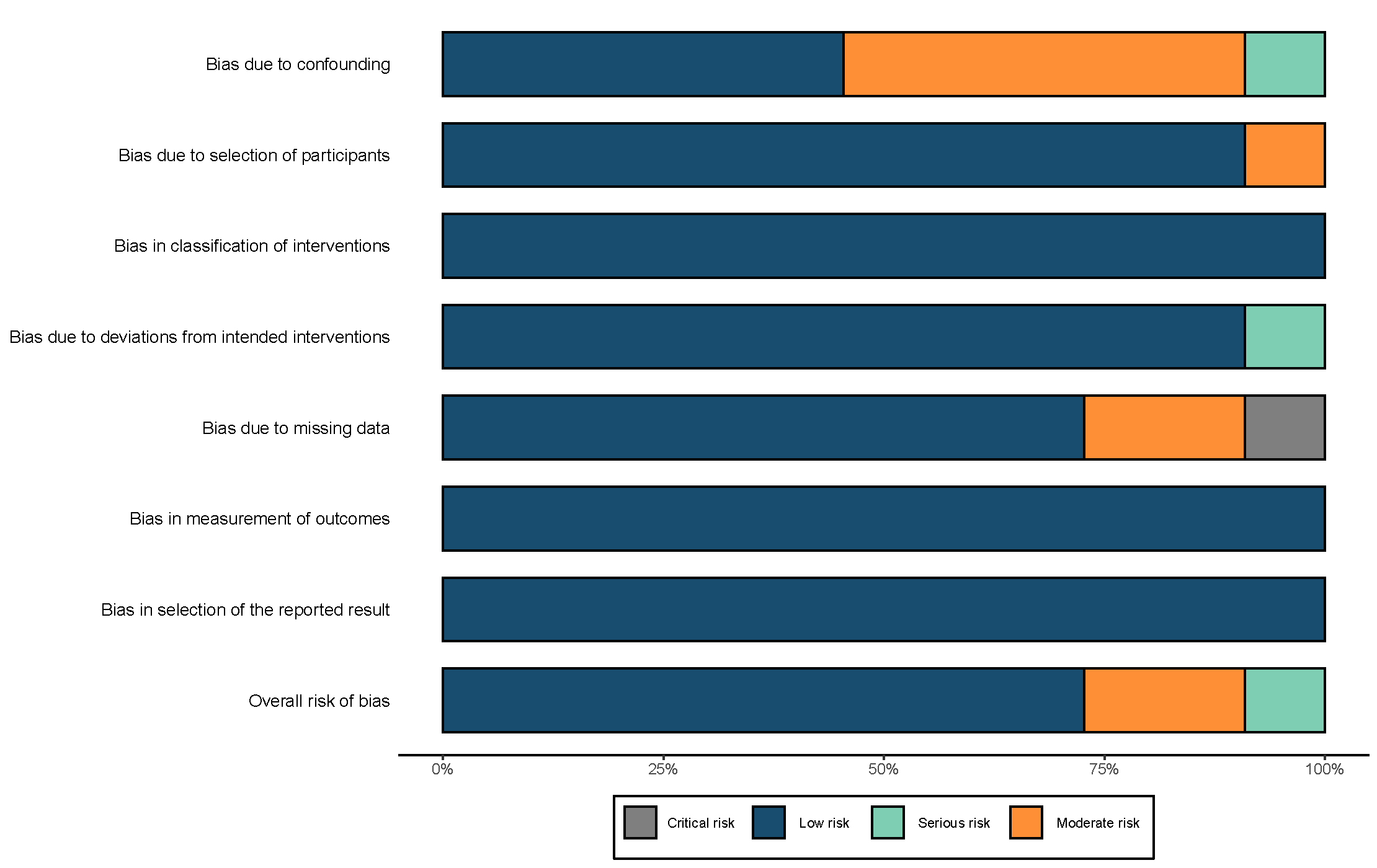


**S1A and S1B Fig. Quality evaluations.** A: Risk biases of each study. S1~S22: the twenty-two studies included in this review, serially. The overall risk was evaluated based on the criteria described previously. B: Summary of risk biases. The length of each color represents the proportion of the risk.


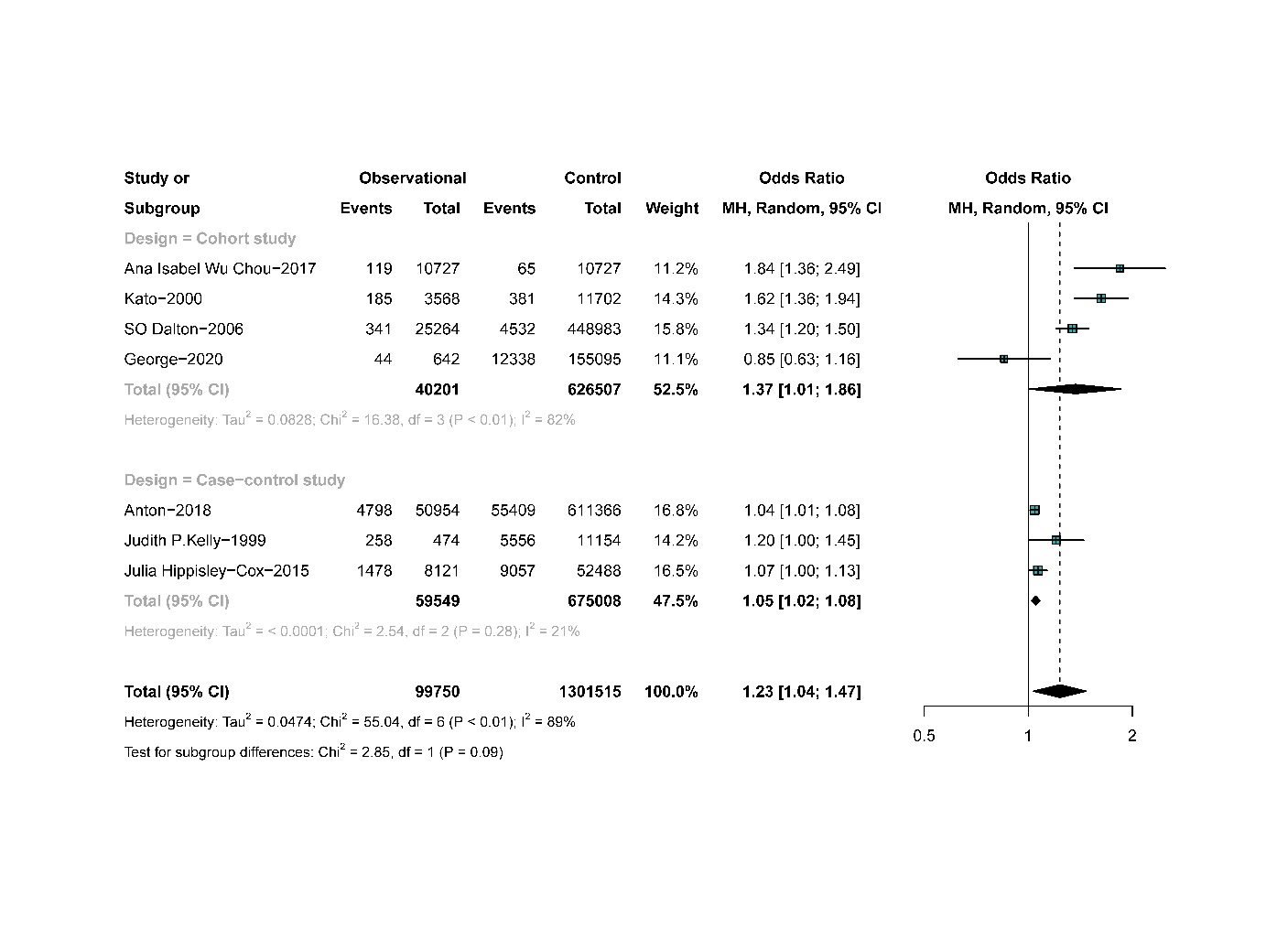


**S2 Fig.** **Subgroup analysis:** **Difference in the prevalence of breast cancer between those exposed to antipsychotics or not in different study designs.** Point sizes are an inverse function of the precision of the estimates, and bars correspond to 95% CIs.


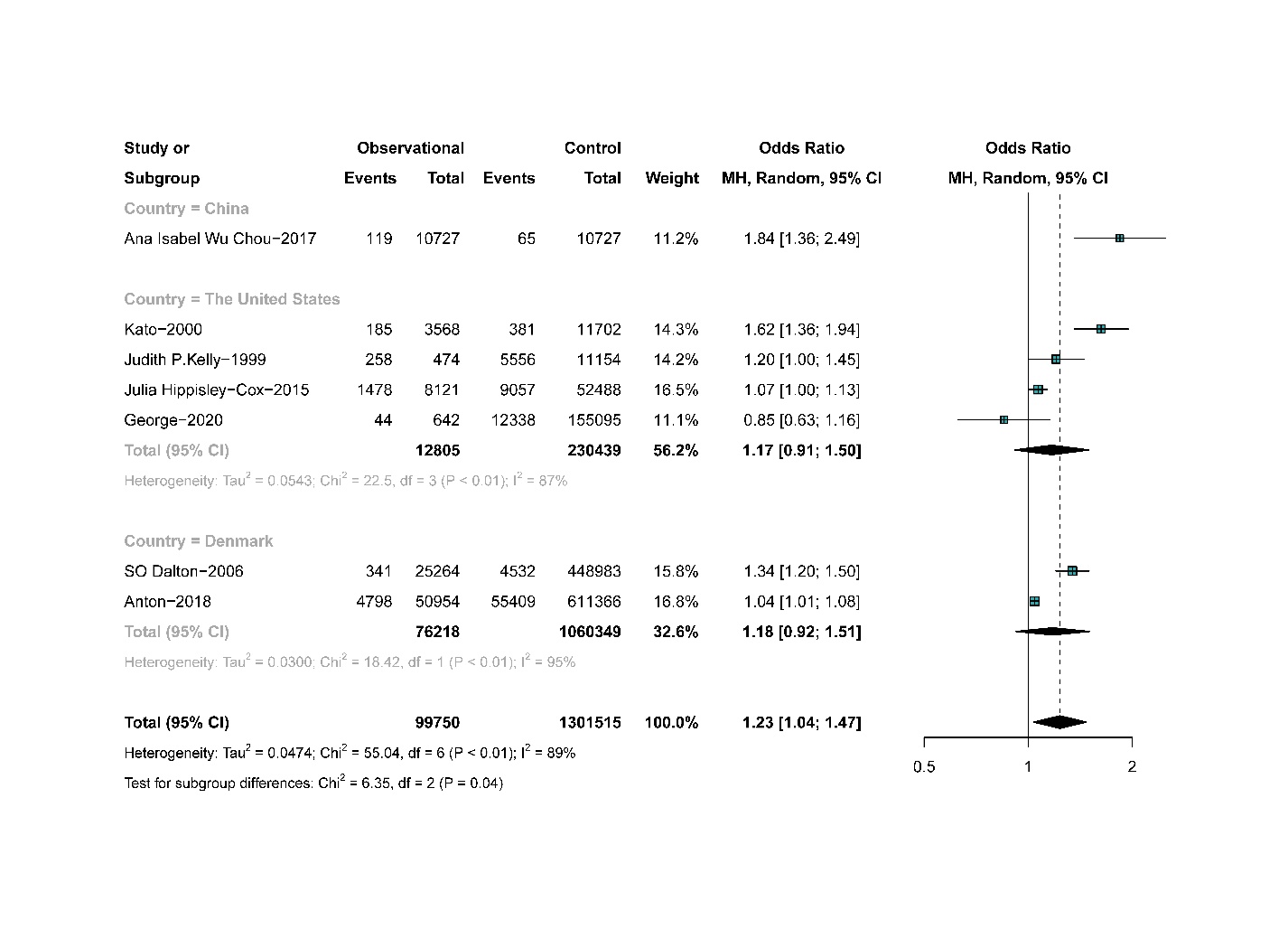


**S3 Fig.** **Subgroup analysis: difference in the prevalence of breast cancer between those exposed to antipsychotics or not in different countries.** Point sizes are an inverse function of the precision of the estimates, and bars correspond to 95% CIs.


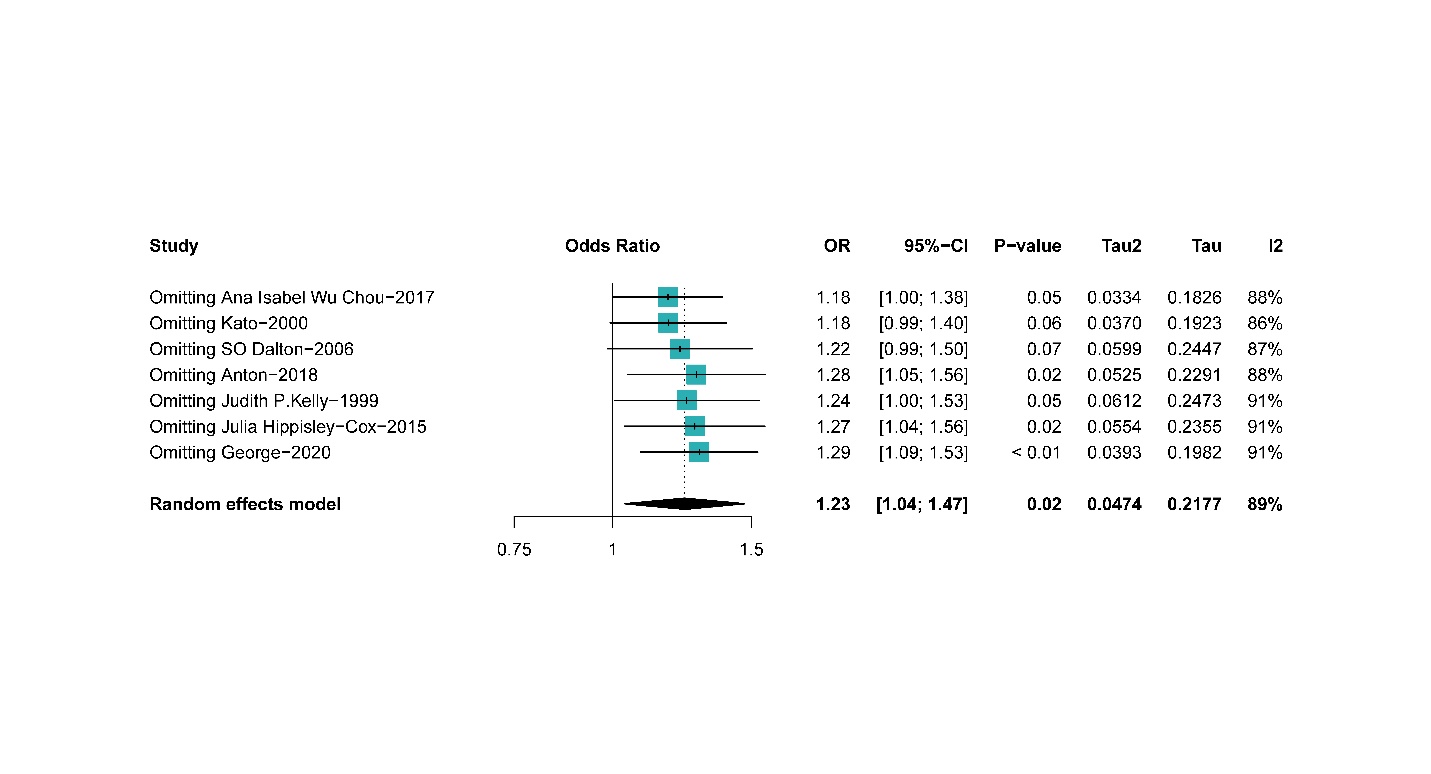


**S4 Fig**. **Sensitivity analysis of comparison A.** Omitting: study number. Data was calculated in a random-effects model.


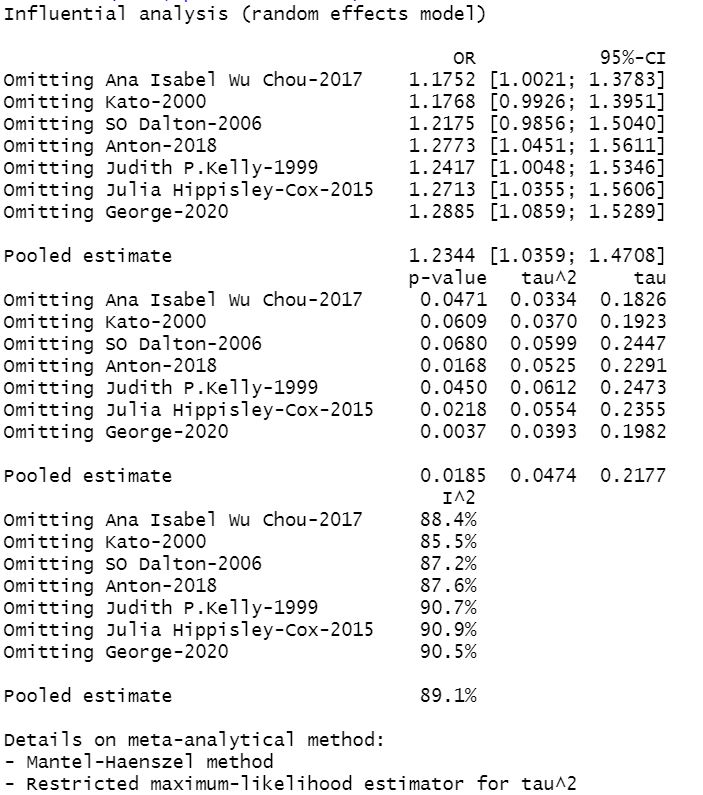


**S5 Fig. Sensitivity analysis of comparison A in R workstation.**
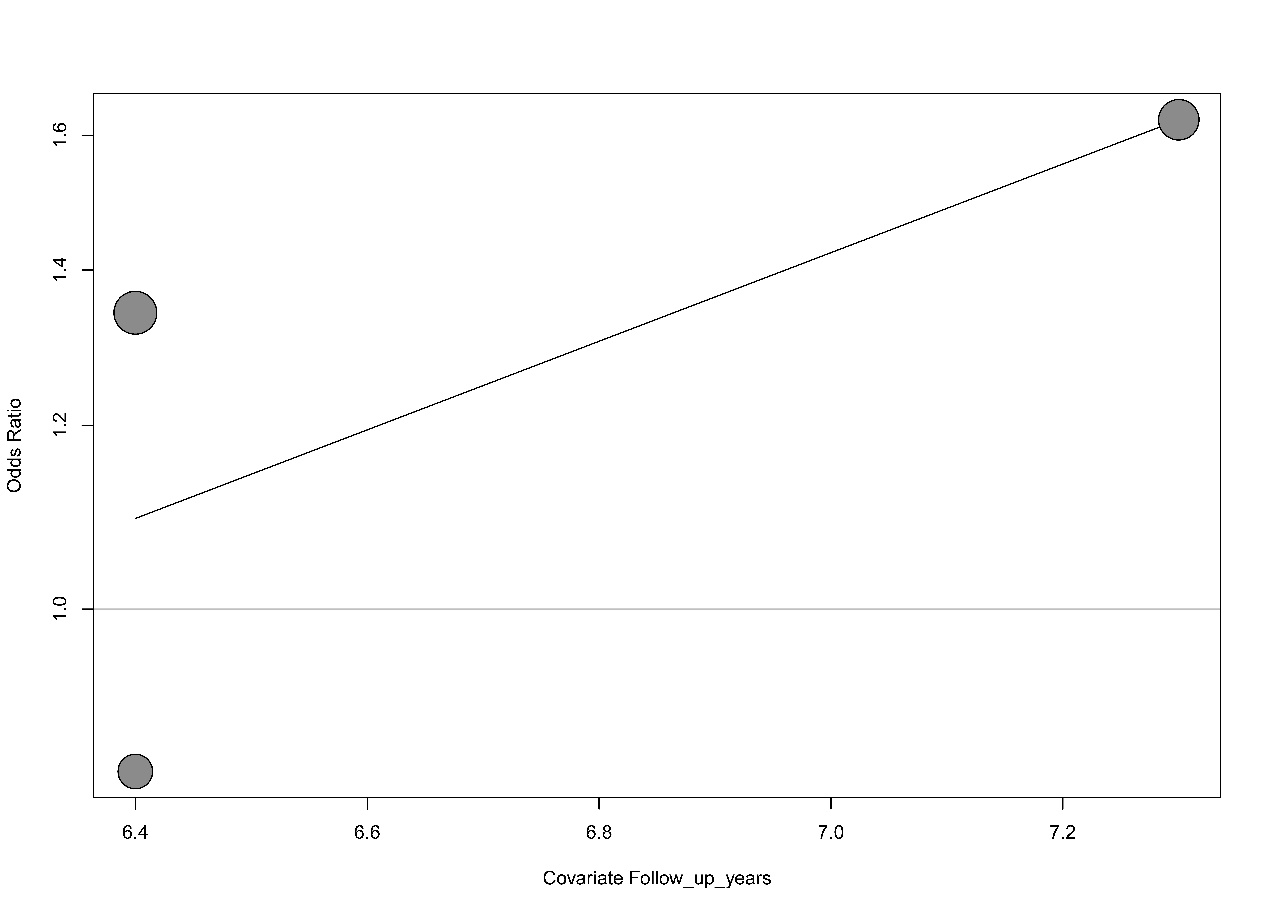
**S6 Fig.** **Meta-regression curve of the follow-up years in comparison A.**


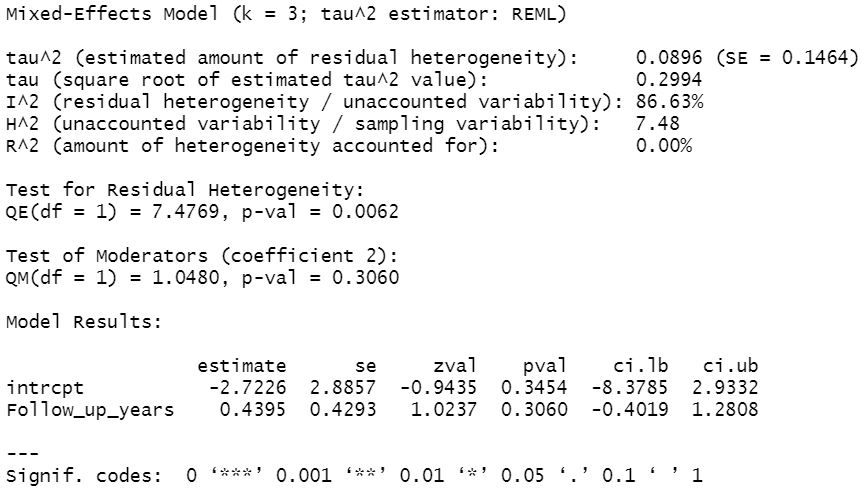
**S7 Fig.** **Meta-regression analysis of the follow-up years in comparison A.**


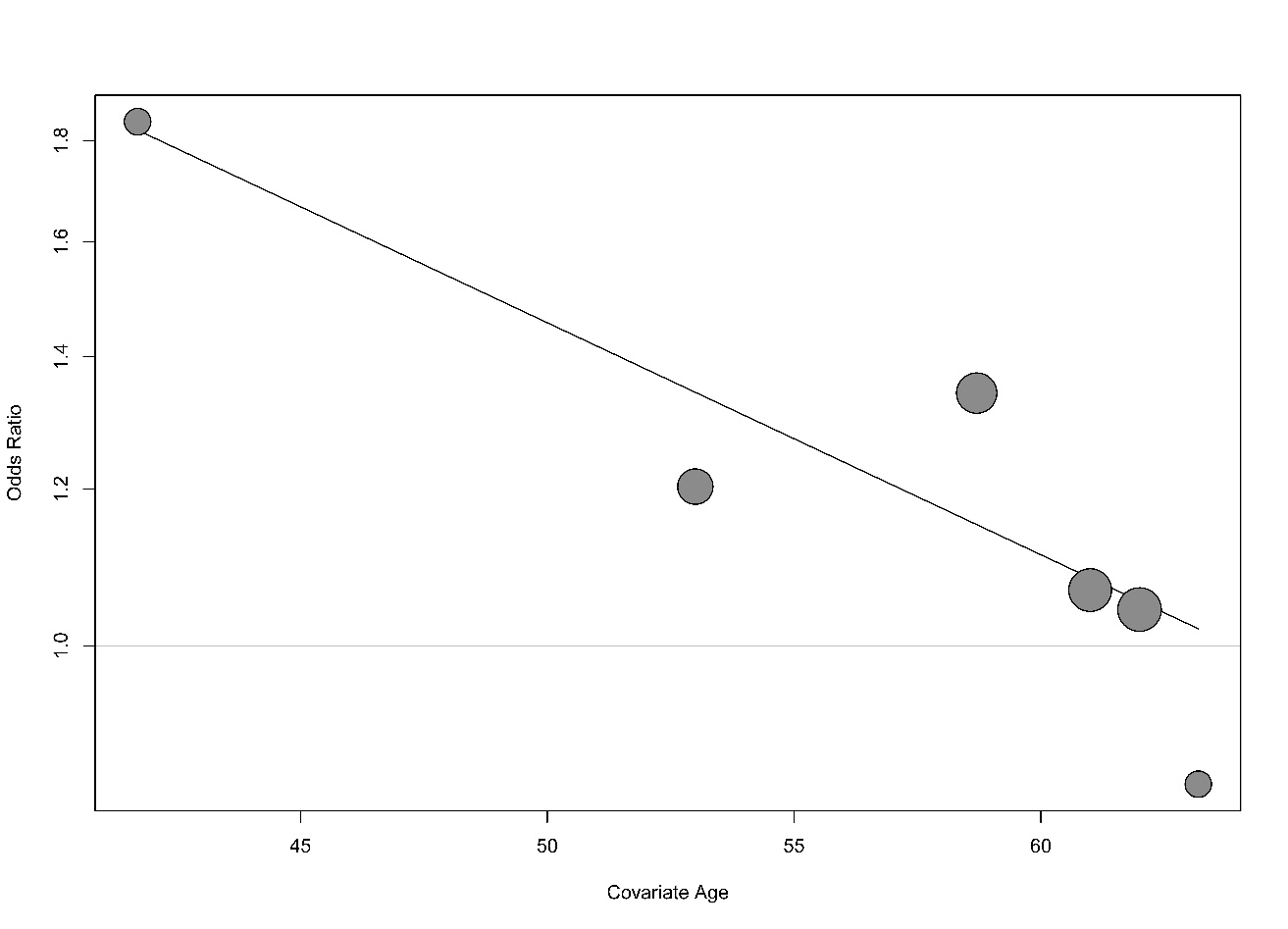
 **S8 Fig.** **Meta-regression curve of the mean age at entry (years) in comparison A.**


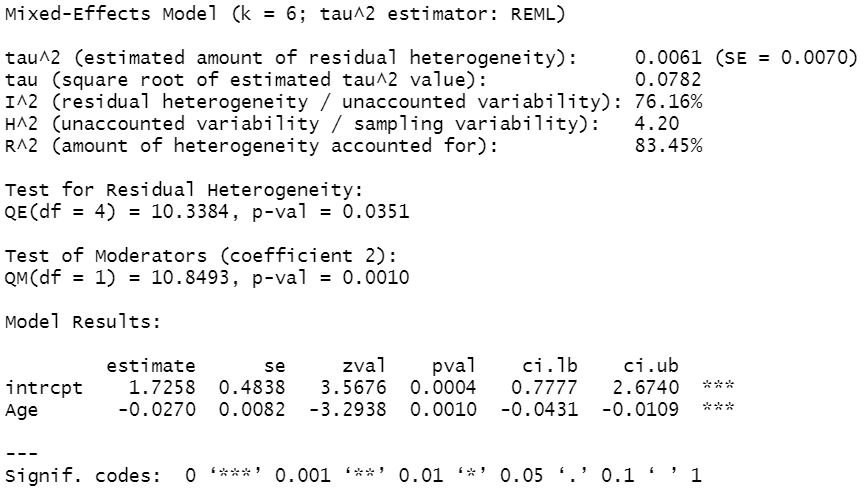
**S9 Fig.** **Meta-regression analysis of the mean age at entry (years) in comparison A.**

**
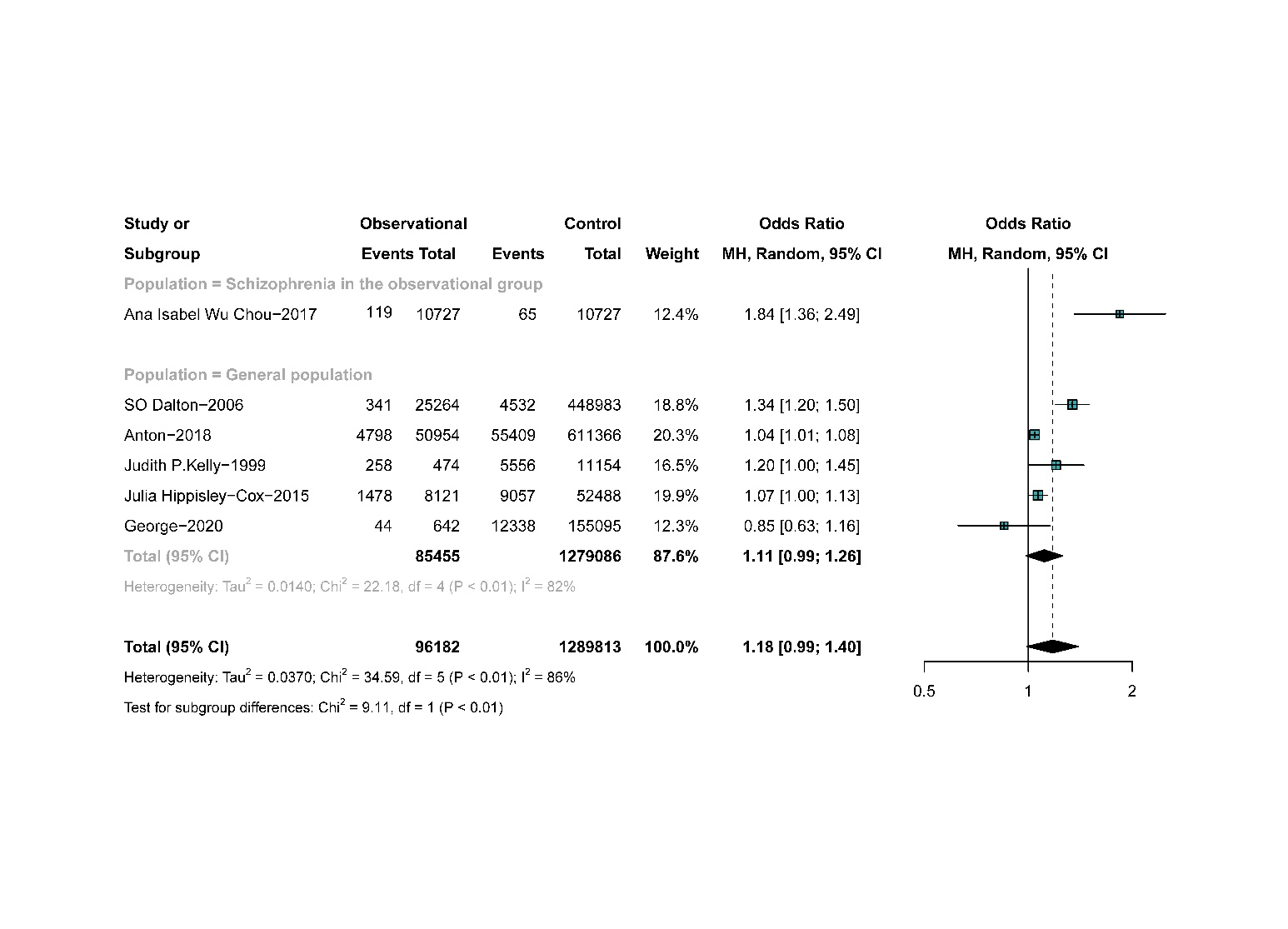
**

**S10 Fig. Subgroup analysis of individuals difference in comparison A.**


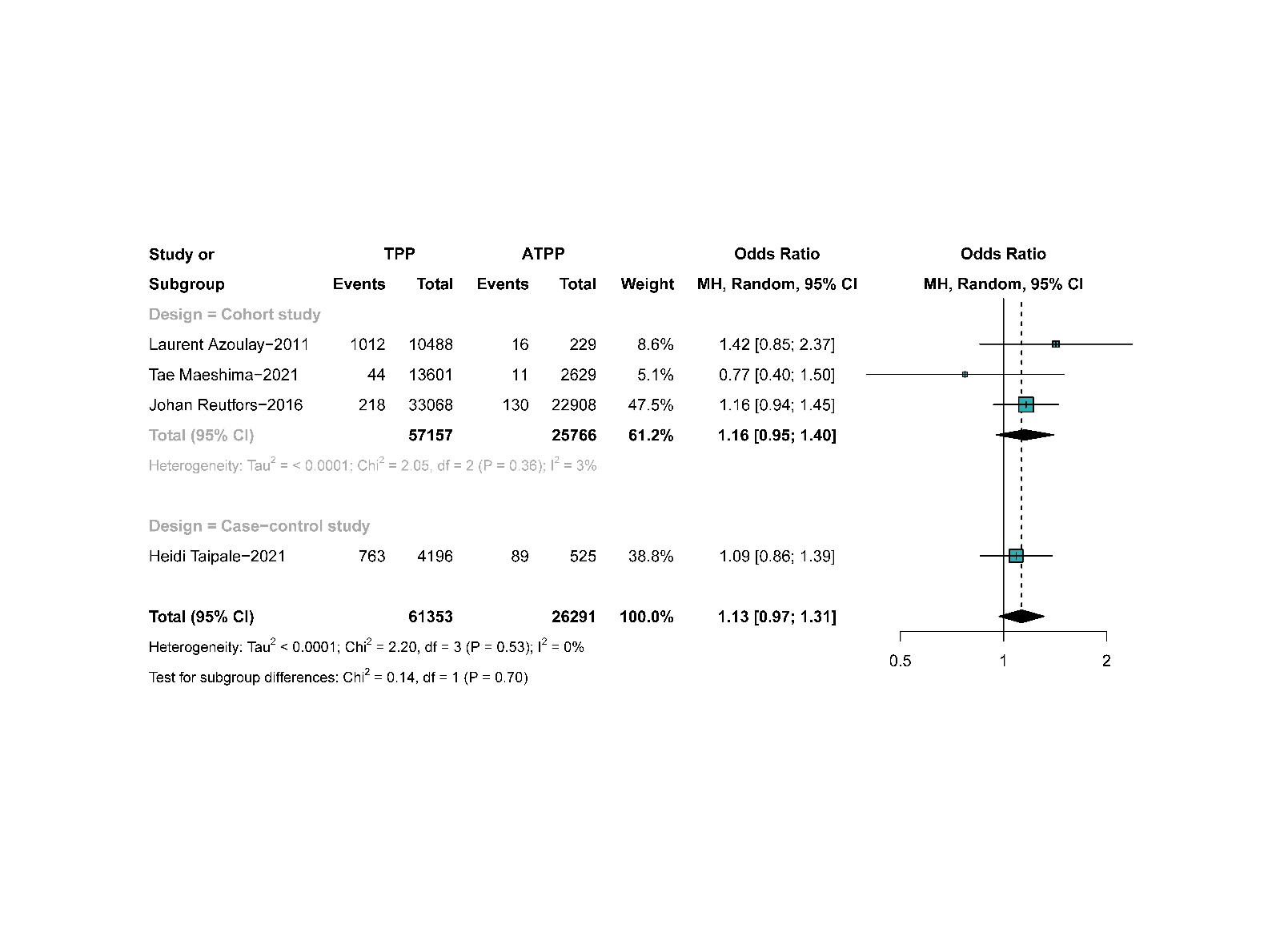
**S11 Fig.** **Subgroup analysis: difference in the prevalence of breast cancer between those exposed to typical or atypical antipsychotics in different study designs.** Point sizes are an inverse function of the precision of the estimates, and bars correspond to 95% CIs.


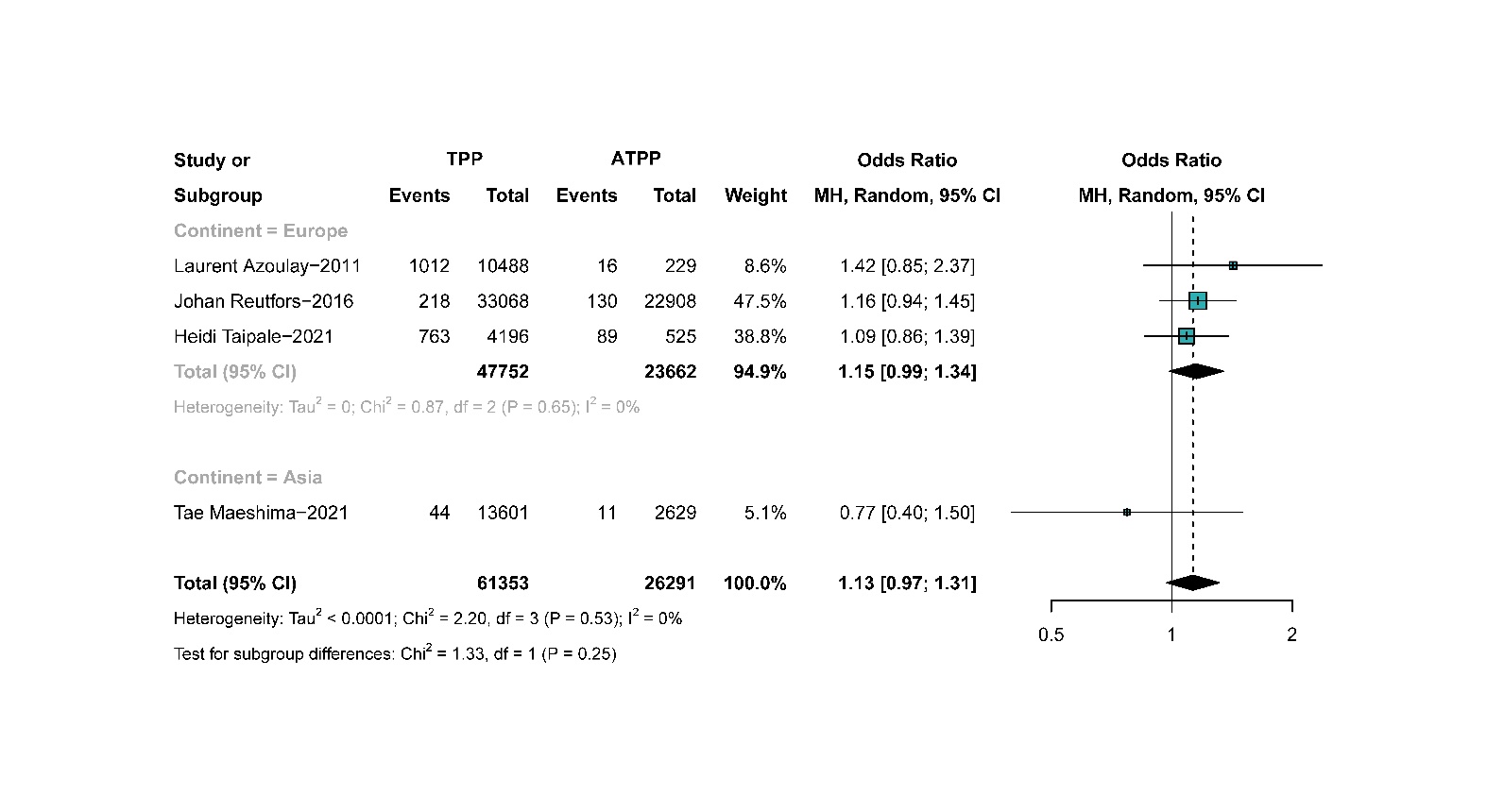
**S12 Fig.** **Subgroup analysis: difference in the prevalence of breast cancer between those exposed to typical or atypical antipsychotics in different continents.** Point sizes are an inverse function of the precision of the estimates, and bars correspond to 95% CIs.


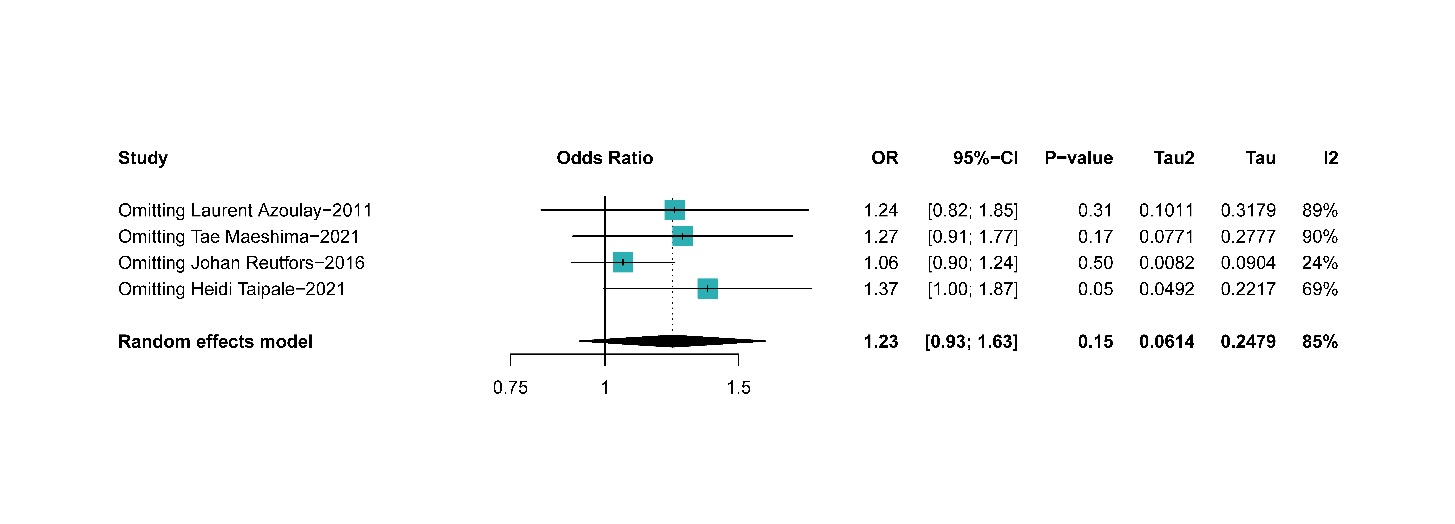
**S13 Fig. Sensitivity analysis of comparison B.** Omitting: study number. Data was calculated in a random-effects model.


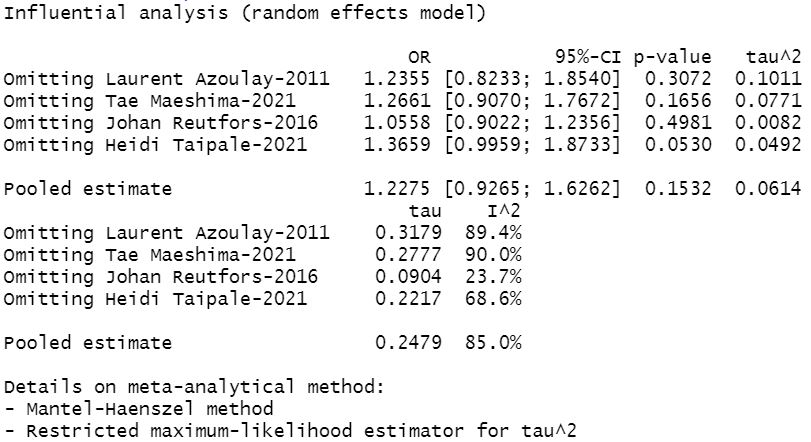
 **S14 Fig.** **Sensitivity analysis of comparison B in R workstation.**


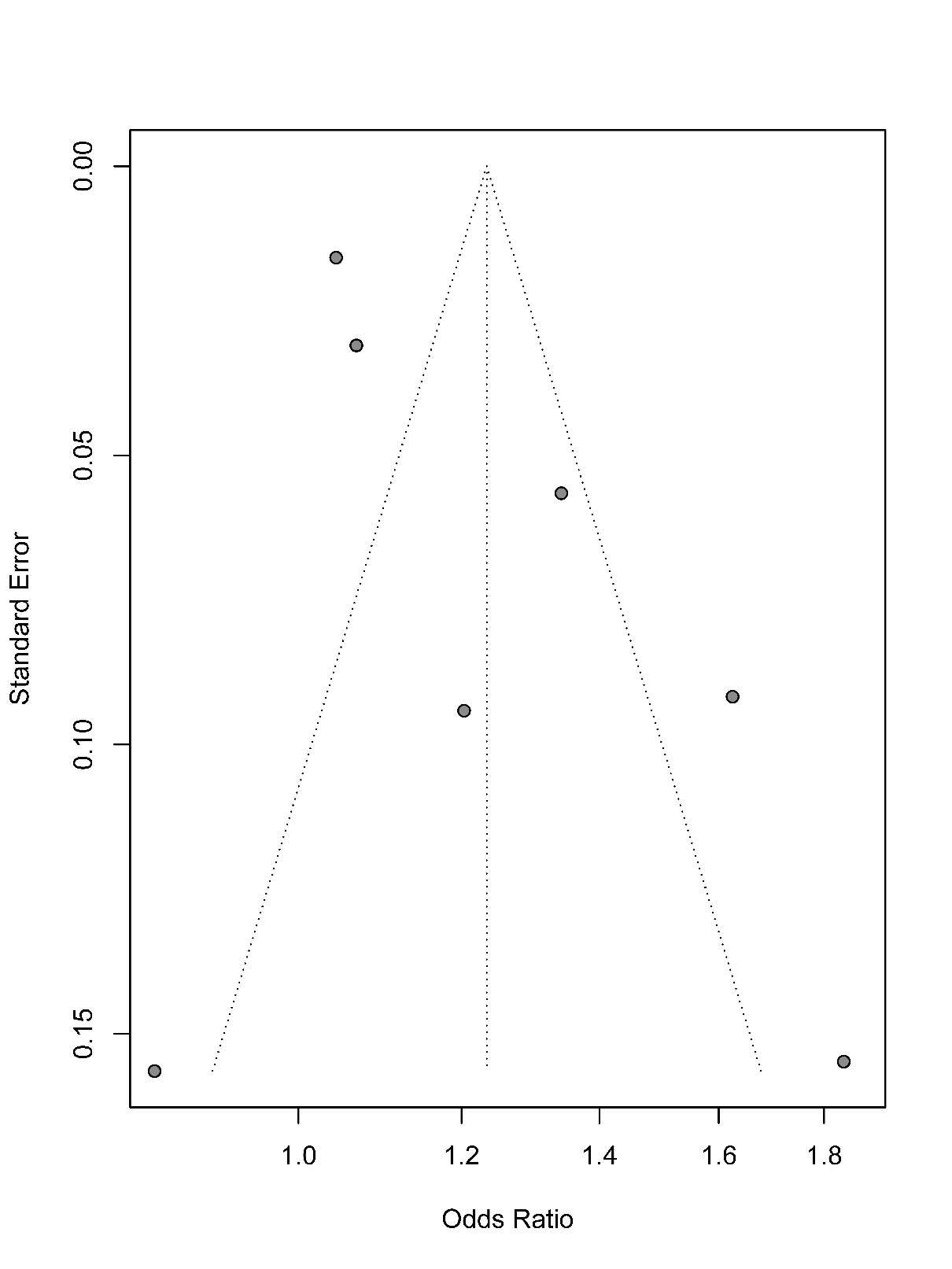


**S15 Fig.** **Deek’s funnel plot of comparison A.**


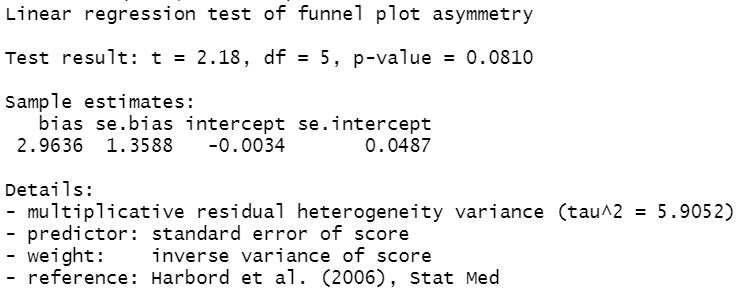


**S16 Fig.** **Peter’s test for comparison A.**


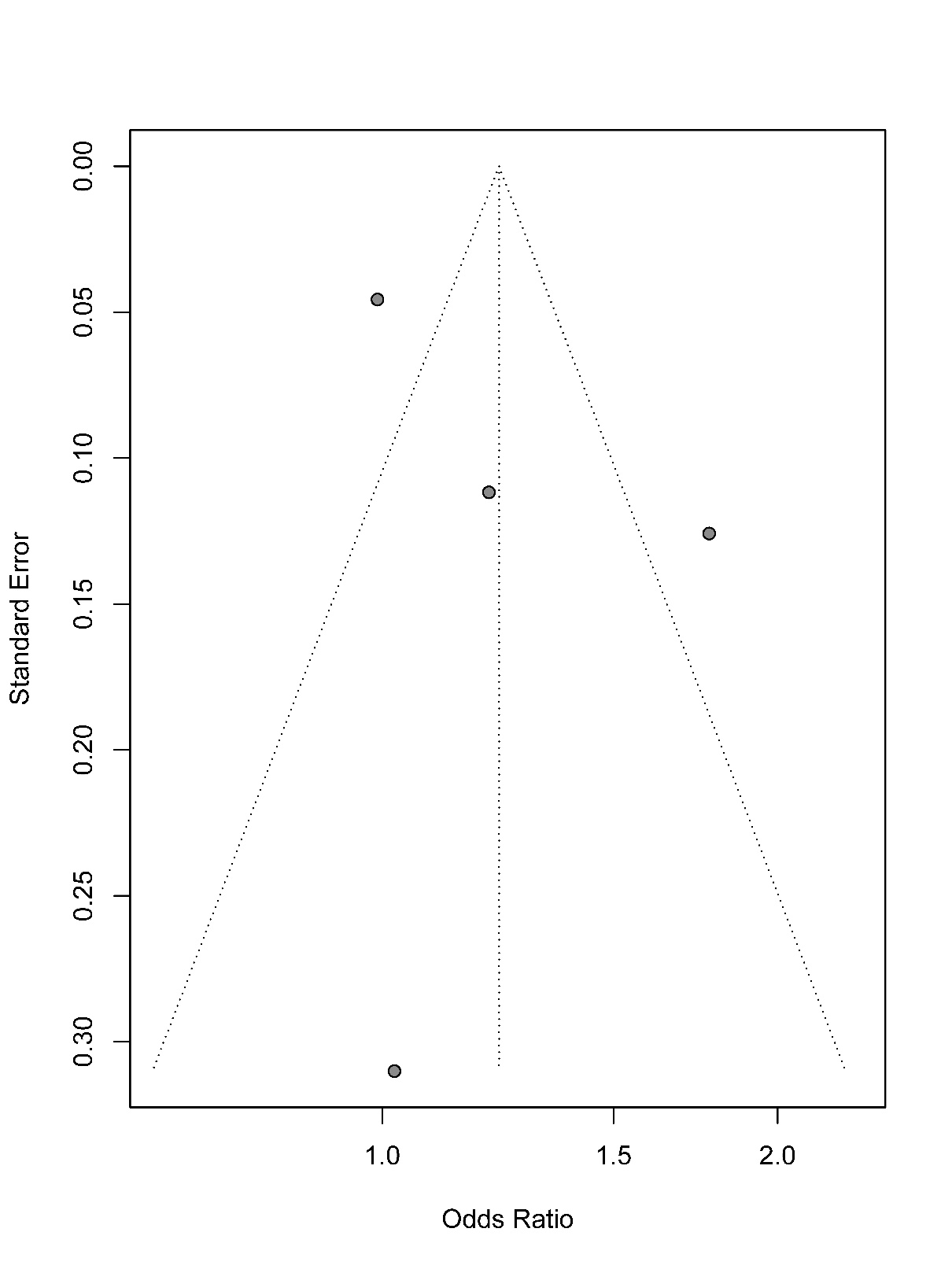


**S17 Fig.** **Deek’s funnel plot of comparison B.**


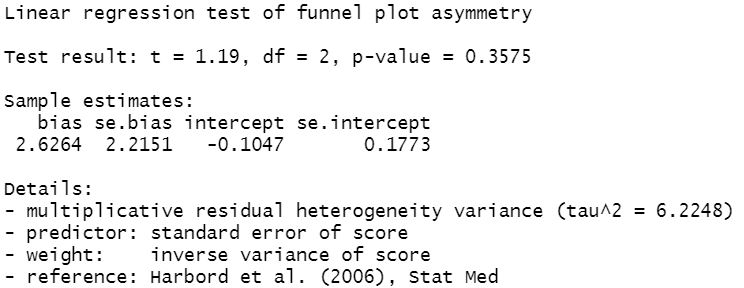


**S18 Fig.** **Peter’s test for comparison B.**


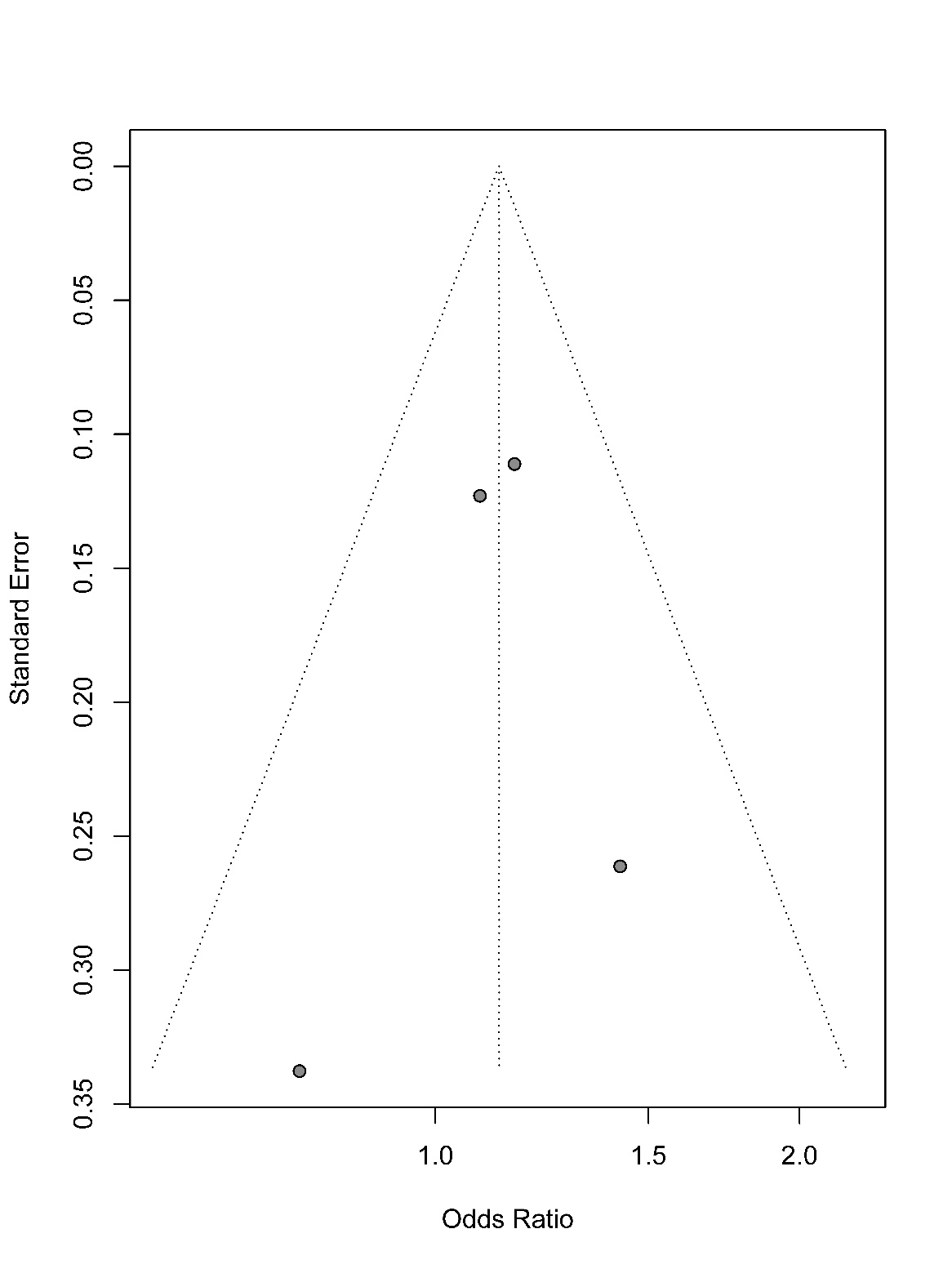


**S19 Fig.** **Deek’s funnel plot of comparison C.**


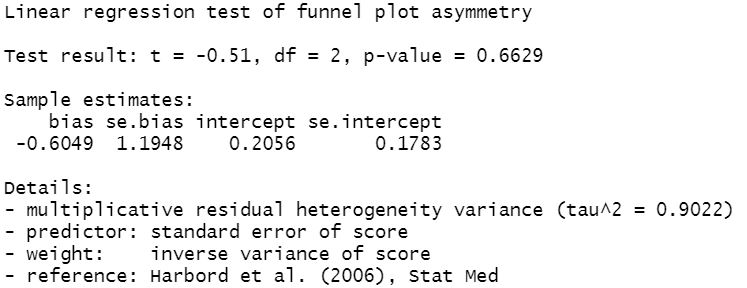
**S20 Fig**. **Peter’s test for comparison C.**


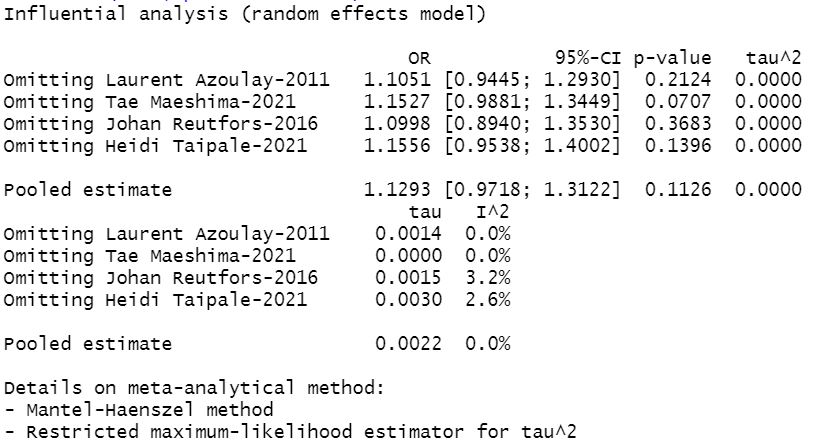


**S21 Fig.** **Sensitivity analysis of comparison C in R workstation.**

**
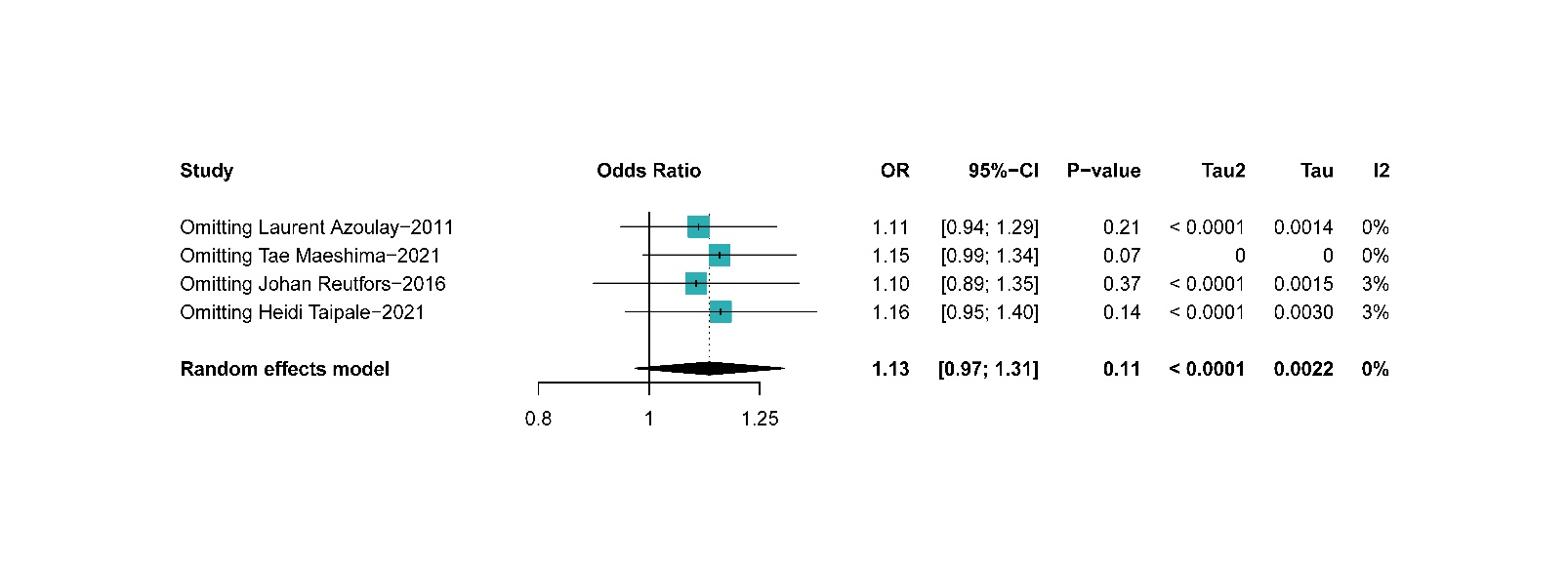
S22 Fig.** **Sensitivity analysis of comparison C.** Omitting: study number. Data was calculated in a random-effects model.

**S1 Appendix. Search strategy in online databases**

| **Number** | **Terms and strategies** | **Logic** |
| --- | --- | --- |
| #1 | Breast Neoplasm | (All Fields/Topic/Theme) |
| #2 | Neoplasm, Breast | (All Fields/Topic/Theme) |
| #3 | Neoplasms, Breast | (All Fields/Topic/Theme) |
| #4 | Breast Tumors | (All Fields/Topic/Theme) |
| #5 | Breast Tumor | (All Fields/Topic/Theme) |
| #6 | Tumor, Breast | (All Fields/Topic/Theme) |
| #7 | Tumors, Breast | (All Fields/Topic/Theme) |
| #8 | Breast Cancer | (All Fields/Topic/Theme) |
| #9 | Cancer, Breast | (All Fields/Topic/Theme) |
| #10 | Malignant Tumor of Breast | (All Fields/Topic/Theme) |
| #11 | Breast Malignant Tumor | (All Fields/Topic/Theme) |
| #12 | Breast Malignant Tumors | (All Fields/Topic/Theme) |
| #13 | Cancer of the Breast | (All Fields/Topic/Theme) |
| #14 | Cancer of Breast | (All Fields/Topic/Theme) |
| #15 | Malignant Neoplasm of Breast | (All Fields/Topic/Theme) |
| #16 | Breast Malignant Neoplasm | (All Fields/Topic/Theme) |
| #17 | Breast Malignant Neoplasms | (All Fields/Topic/Theme) |
| #18 | Mammary Cancer | (All Fields/Topic/Theme) |
| #19 | Cancer, Mammary | (All Fields/Topic/Theme) |
| #20 | Cancers, Mammary | (All Fields/Topic/Theme) |
| #21 | Mammary Cancers | (All Fields/Topic/Theme) |
| #22 | Mammary Carcinoma, Human | (All Fields/Topic/Theme) |
| #23 | Carcinoma, Human Mammary | (All Fields/Topic/Theme) |
| #24 | Carcinomas, Human Mammary | (All Fields/Topic/Theme) |
| #25 | Human Mammary Carcinomas | (All Fields/Topic/Theme) |
| #26 | Mammary Carcinomas, Human | (All Fields/Topic/Theme) |
| #27 | Human Mammary Carcinoma | (All Fields/Topic/Theme) |
| #28 | Mammary Neoplasms, Human | (All Fields/Topic/Theme) |
| #29 | Human Mammary Neoplasm | (All Fields/Topic/Theme) |
| #30 | Human Mammary Neoplasms | (All Fields/Topic/Theme) |
| #31 | Neoplasm, Human Mammary | (All Fields/Topic/Theme) |
| #32 | Neoplasms, Human Mammary | (All Fields/Topic/Theme) |
| #33 | Mammary Neoplasm, Human | (All Fields/Topic/Theme) |
| #34 | Breast Carcinoma | (All Fields/Topic/Theme) |
| #35 | Breast Carcinomas | (All Fields/Topic/Theme) |
| #36 | Carcinoma, Breast | (All Fields/Topic/Theme) |
| #37 | Carcinomas, Breast | (All Fields/Topic/Theme) |
| #38 | #1 OR #2 OR #3 OR #4 OR #5 OR #6 OR #7 OR #8 OR #9 OR #10 OR #11 OR #12 OR #13 OR #14 OR #15 OR #16 OR #17 OR #18 OR #19 OR #20 OR #21 OR #22 OR #23 OR #24 OR #25 OR #26 OR #27 OR #28 OR #29 OR #30 OR #31 OR #32 OR #33 OR #34 OR #35 OR #36 OR #37 | |
| #39 | Neuroleptics | (All Fields/Topic/Theme) |
| #40 | Antipsychotic Medication | (All Fields/Topic/Theme) |
| #41 | Medication, Antipsychotic | (All Fields/Topic/Theme) |
| #42 | Neuroleptic Agent | (All Fields/Topic/Theme) |
| #43 | Agent, Neuroleptic | (All Fields/Topic/Theme) |
| #44 | Neuroleptic Drug | (All Fields/Topic/Theme) |
| #45 | Drug, Neuroleptic | (All Fields/Topic/Theme) |
| #46 | Neuroleptic | (All Fields/Topic/Theme) |
| #47 | Antipsychotic | (All Fields/Topic/Theme) |
| #48 | Antipsychotic Drugs | (All Fields/Topic/Theme) |
| #49 | Antipsychotics | (All Fields/Topic/Theme) |
| #50 | Major Tranquilizers | (All Fields/Topic/Theme) |
| #51 | Neuroleptic Agents | (All Fields/Topic/Theme) |
| #52 | Tranquilizing Agents, Major | (All Fields/Topic/Theme) |
| #53 | Major Tranquilizing Agents | (All Fields/Topic/Theme) |
| #54 | Tranquillizing Agents, Major | (All Fields/Topic/Theme) |
| #55 | Major Tranquillizing Agents | (All Fields/Topic/Theme) |
| #56 | Major Tranquilizer | (All Fields/Topic/Theme) |
| #57 | Tranquilizer, Major | (All Fields/Topic/Theme) |
| #58 | Neuroleptic Drugs | (All Fields/Topic/Theme) |
| #59 | Antipsychotic Drug | (All Fields/Topic/Theme) |
| #60 | Drug, Antipsychotic | (All Fields/Topic/Theme) |
| #61 | Antipsychotic Agent | (All Fields/Topic/Theme) |
| #62 | Agent, Antipsychotic | (All Fields/Topic/Theme) |
| #63 | Antipsychotic Effect | (All Fields/Topic/Theme) |
| #64 | Effect, Antipsychotic | (All Fields/Topic/Theme) |
| #65 | Antipsychotic Effects | (All Fields/Topic/Theme) |
| #66 | #39 OR #40 OR #41 OR #42 OR #43 OR #44 OR #45 OR #46 OR #47 OR #48 OR #49 OR #50 OR #51 OR #52 OR #53 OR #54 OR #55 OR #56 OR #57 OR #58 OR #59 OR #60 OR #61 OR #62 OR #63 OR #64 OR #65 | |
| #67 | #38 AND #66 |  |
